# The Biology and Ecology of Cat Fleas and Advancements in Their Pest Management: A Review

**DOI:** 10.3390/insects8040118

**Published:** 2017-10-27

**Authors:** Michael K. Rust

**Affiliations:** Department of Entomology, University of California Riverside, Riverside, CA 92521, USA; michael.rust@ucr.edu; Tel.: +1-951-827-5327

**Keywords:** *Ctenocephalides felis felis*, systemic insecticides, insect growth regulators, insecticide resistance

## Abstract

The cat flea *Ctenocephalides felis felis* (Bouché) is the most important ectoparasite of domestic cats and dogs worldwide. It has been two decades since the last comprehensive review concerning the biology and ecology of *C. f. felis* and its management. Since then there have been major advances in our understanding of the diseases associated with *C. f. felis* and their implications for humans and their pets. Two rickettsial diseases, flea-borne spotted fever and murine typhus, have been identified in domestic animal populations and cat fleas. Cat fleas are the primary vector of *Bartonella henselae* (cat scratch fever) with the spread of the bacteria when flea feces are scratched in to bites or wounds. Flea allergic dermatitis (FAD) common in dogs and cats has been successfully treated and tapeworm infestations prevented with a number of new products being used to control fleas. There has been a continuous development of new products with novel chemistries that have focused on increased convenience and the control of fleas and other arthropod ectoparasites. The possibility of feral animals serving as potential reservoirs for flea infestations has taken on additional importance because of the lack of effective environmental controls in recent years. Physiological insecticide resistance in *C. f. felis* continues to be of concern, especially because pyrethroid resistance now appears to be more widespread. In spite of their broad use since 1994, there is little evidence that resistance has developed to many of the on-animal or oral treatments such as fipronil, imidacloprid or lufenuron. Reports of the perceived lack of performance of some of the new on-animal therapies have been attributed to compliance issues and their misuse. Consequentially, there is a continuing need for consumer awareness of products registered for cats and dogs and their safety.

## 1. Introduction

The cat flea, *Ctenocephalides felis felis* (Bouché), is the most important ectoparasite of domesticated cats and dogs worldwide. The last comprehensive reviews of the biology and control of the cat flea were provided two decades ago [1,2]. Several reviews dealing with insecticide resistance, toxicology of veterinary insecticides, and the control of cat fleas have been written during this period. This systematic review will incorporate them, the advancements in our knowledge about cat flea biology, ecology, and the rapidly changing control strategies over the past 20 years. In some cases, non-English articles have not been cited because their abstracts were not-detailed enough to be informative and others could not be obtained. The following databases were consulted for articles appearing from 1996 to 2017: BIOSIS Previews, Google Scholar, PubMed, Web of Science, and Zoological Record. Of the 478 articles reviewed, the distribution of references in the sections covered is approximately as follows: Biology and Ecology (134), Veterinary and Medical Importance (54), Rearing and Testing Methodologies (15), Pest Management (221), Environmental Control (5), Toxicology of Ecotoparasiticides (27), Treatment Failure and Insecticide Resistance (18), Natural and Biological Control (3), and IPM (3).

## 2. Cat Flea Biology and Ecology

Several general reviews of *C. f. felis* biology have been published since 1997 [3,4,5,6,7,8,9,10,11]. Our understanding regarding the geographical distribution of *C. f. felis* and its alternate hosts continues to expand. *C. f. felis* is truly a global pest and global warming will probably not affect the distribution of cat fleas. The low outside persistence of *C. f. felis*, indoor breeding sites, a highly specialized life cycle, and a need for specific temperature and humidity conditions for development are all factors that suggest the distribution of cat fleas will remain the same [12]. However, with increased temperatures, the number of generations per year and potential density of cat fleas might dramatically increase.

Cat fleas belong to the Order Siphonaptera and the family Pulicidae. Within the family Pulicidae, the genus *Ctenocephalides* has undergone some major revisions with the advent of molecular systematics and critical reviews of existing morphological characters. Characters on the aedeagus such as the hamulus, lobes and tubus interior permit the identification of most of the species of *Ctenocephalides* [13]. However, the existence of morphological variations of characters used to differentiate *C. f. felis* and *C. canis* require that host data, geographical distribution, and the prevalence of infestations also be used in their determination [14,15]. From a systematic perspective, four subspecies of cat fleas had existed for six decades; namely, *C. felis damarensis*, *C. felis felis*, *C. felis orientis*, *and C. felis strongylus* [16]. ITS1 and ITYS2 nucleotide sequences and 16SrDNA sequences were invariant in a number of *C. felis* populations collected worldwide and overall findings did not support subspecies of *C. felis* [17]. Several microsatellites have been identified that could help determine if host specific strains of *C. f. felis* exist, the existence of subspecies, and detailed epidemiological studies of *Rickettsia felis* [18]. Sequences of cytochrome c oxidase subunits *cox1* and *cox 2* indicate that *C. f. felis* and *C. f. strongylus* are paraphyletic and *C. f. orientis* is monophyletic [19]. Three distinct clades of *C. f. felis* were found. Similar studies with subunits *cox1* and *cox2* revealed that *C. f. felis* from New Zealand belonged to Clade 1 like those of Australia and Europe [20]. No intraspecific variation was found at the ITS1 marker for 52 *C. f. felis* specimens analyzed from 17 different locations in south central US, suggesting either a genetic bottleneck or that they were recently introduced [21]. Populations of *C. f. felis* and *C. canis* from Spain, Iran and South Africa were examined and ITS1 sequences conducted. Both species were clearly separated [22]. A matrix-assisted laser desorption/ionization time-of-flight mass spectrometry (MALDI-TOF MS) technique was used to identify important pest species of fleas. A single fresh specimen provided unequivocal identification to species. Specimens preserved in ethanol provided variable results depending upon the length of time in ethanol [23].

Recent systematic efforts including molecular techniques have elevated two of the subspecies to full species, *C. damarensis* and *C. orientis* [13,24]. *C. f. felis* was found only on cats and dogs whereas *C. f. strongylus* was only found on large farm animals in Libya [25]. In South Africa, *C. f. strongylus* has been collected on the wild cat *Caracal caracal* and domestic dogs in rural areas [26]. Possibly *C. f. strongylus* will also be elevated to species status in the future.

For brevity *C. felis felis* will be referred to as *C. felis*.

### 2.1. Geographical Distribution and Hosts

Numerous surveys of the ectoparasites of companion animals have been conducted worldwide and they are briefly reviewed in order according to the continent, region and country. A review of the fleas of the hosts belonging to family Canidae indicate that *C. felis* is the most common flea of domesticated dogs globally [27]. *C. felis* has been collected on feral animals such as opossums, fox, rats, mongoose, and hedgehogs and this data are summarized in Table 1. In general the numerous reports confirm that cats are more often infested by *C. felis* than dogs; the prevalence of *C. felis* is seasonal, but it appears throughout the year; and female fleas are collected more often than males. *C. canis* is more prevalent on dogs in some countries such as Greece, Iran, and Turkey.

In Nigeria, 200 cats were examined of which 13% had *C. felis* [51]. In Hawassa, Ethiopia, there was a high incidence of ectoparasites on cats and dogs with 67% of cats and 82.9% of dogs infested with *C. felis* [52]. *C. felis* is not common in South Africa, but was taken from a dog in Johannesburg [26].

In a survey of 214 dogs, 110 (51.4%) were seropositive for anti-flea IgE in Japan indicating that dogs had been infested at one time. Dogs in northern areas of Japan thought to be flea free were also seropositive [53]. A survey of 324 stray dogs in India indicated that 24% were infested of which *C. felis* comprised 10.4% [54]. In Thailand, only *C. felis* were found on cats whereas *C. orientis* was found on dogs [55]. Stray cats (*n* = 200) in Taipei were examined and 82% were infested with *C. felis* [56]. A survey of 83 dogs from three regions of Iran resulted in 407 fleas of which 67.5% were *C. felis* [57]. In another study, along the Iraq-Iran border, 802 dogs and 50 cats were surveyed of which 2.4% of dogs and 65% of the cats were infested with *C. felis* [58]. Only 2 of 126 dogs in southwest Iran were infested with *C. felis* [59]. Of the fleas collected from 70 stray dogs in northern and central Iran, 19.9% of them were *C. felis* [60]. In a study in Israel, 340 stray cats were examined of which 54.7% were infested with *C. felis*. Fleas were recovered every month with highest numbers in the autumn [61].

In Australia, 98.8% of the 2500 fleas collected were *C. felis* and a single haplotype of the *cox2* gene sequence was found [62]. In Borneo, 195 dogs were examined and 1965 fleas collected of which 25.4% were *C. felis*, the remaining being *C. orientis* [63]. *C. felis* was collected from both cats and dogs in Guam [64].

Many of the recent surveys have been conducted throughout Europe. A survey of ectoparasites and endoparasites of 1519 cats from seven countries in Europe found that 15.5% of the cats brought to veterinary clinics were infested with fleas. Of the cats suffering from anemia 93.5% were highly infested with fleas [65]. In southern Poland, of the 225 parasitic insects collected from pets only 3 were *C. felis* [66]. When dogs and cats were surveyed in the Czech Republic, 60% were *C. felis*, belonging to the cosmopolitan *cox1* haplotype. A novel haplotype was found in both the Czech Republic and Romania [67]. A survey of 1342 dogs and 1378 cats presented to 22 different clinics in Serbia found that 79.2% of the fleas were *C. felis* with the most being found on cats from July to September [68]. In Hungary, 2267 dogs were inspected of which 115 dogs were infested with *C. felis* and 23 of 100 cats inspected were infested with *C. felis*. Fleas were more prevalent from rural animals than from urban animals [69]. In western Hungary, 71% of the 82 cats examined had *C. felis* [70]. In Turkey, 48 dogs were examined and 43.8% were infested with fleas. *C. felis* was found on 4.2% of the dogs with an average 5 fleas/dog. There were no seasonal patterns [71]. In Tirana, Albania, 128 dogs and 26 cats were examined for ectoparasites of which 5% of the dogs and 100% of the cats were infested with *C. felis*. Fleas were encountered year round [72]. In another study, from Tirana, 131 domestic cats were examined for ectoparasites with 52% being infested with *C. felis*. *C. felis* were collected year round with 48.8% being taken in the autumn from September to November [73]. Four clinics in Albania examined 602 client-owned dogs and found that 3.0% were infested with *C. felis* [74]. A survey in Germany found that 5.1% of dogs and 14.3% of cats were infested with fleas. Of the fleas collected, 81.1% were *C. felis* and there were no differences in urban vs. rural infestation rates [75]. Another survey in Germany found that 71.1% of dogs and 83.5% of cats were infested with *C. felis*. The increased prevalence over last decades may be due to temperature controlled housing [35]. In France, 392 dogs infested with fleas were examined and 86.6% of the fleas were *C. felis*. *C. felis* were found throughout France indoors and outdoors. Of the fleas collected from dogs living at elevations >400 m only 11.2% were *C. felis* compared with 32.5% *C. canis* [76].

Thirty-one clinics in the UK were surveyed and a total of 2653 dogs and 1508 cats were examined of which 21.1% of the cats and 6.8% of the dogs were infested. *C. felis* was the most common flea with 98.9% on cats and 93.2% on dogs [77]. Of the 138 fleas collected in the UK in the autumn and winter, 96% were *C. felis*. The adult female cat fleas continued to mature oocytes throughout the fall and winter [78].

In Serbia, of the 1484 dogs brought to clinics from several cities 26.3% were infested with fleas of which 71.9% were *C. felis*. The highest infestation rates were from June to October [79]. In Greece, 13.7% of fleas collected from dogs were *C. felis*, the remaining being *C. canis* [80]. A survey in southern Italy found *C. felis* on 16.3% of the 1376 dogs examined [81]. Fleas were detected throughout the year with the greatest prevalence being between June and October. In another study in Italy, 80.3% of the fleas collected from 73 dogs and 44 cats were *C. felis* [82]. Of the 3032 fleas collected from 1084 dogs from 42 locations in Spain, 81.7% were *C. felis*. *C. felis* were most abundant in early summer and late autumn [83]. In another survey from Spain, 77 veterinary clinics collected 1938 fleas from 217 cats of which 98.4% were *C. felis*. There were lower infestation rates in warm summer months and overall flea abundance was positively associated with rainfall [84]. In Greece, 341 stray cats were examined and 24.3% were infested with *C. felis*. Cats with long hair (>4 cm long) had significantly more ectoparasites than did short haired cats with 42.3% of the ectoparasites being *C. felis* [85]. Of 242 stray dogs examined, 46.2% were infested with either *C. felis* or *C. canis* in Greece [86].

In North America, a survey of 200 feral cats from north central Florida found that 92.5% of them were infested with *C. felis*. The highest flea counts were in June and July (16.6–18.3 fleas per cat) and the lowest from August to September (7.7 to 8.4 fleas per cat) [87]. *C. felis* was reported from dogs in South Carolina [46]. In Georgia, 2518 fleas were collected from dogs of which 61% were *C. felis*. Three female fleas were collected for each male. The vast majority of fleas were collected from August through October [88]. Of 673 free-roaming cats examined in the central US, 71.6% had fleas of which 97.2% were *C. felis* [89]. *C. felis* is common and widespread flea of pets in West Virginia and Virginia. Fleas were found in every month with June, September and October being the highest and April the lowest [44,45]. In Mexico, about 30% of the dogs (1803) and cats (517) examined were infested with fleas. Of the 4215 fleas collected, 81.1% were *C. felis*. There were no seasonal variations in the flea prevalence [45]. Similarly, of the 358 cats included in another study 53% were infested with fleas. Of the 2985 fleas collected, 89% were *C. felis* [90]. In Aguasclientes, Mexico, 863 dogs were examined and 38% of the 629 fleas collected were *C. felis* with the higher prevalence in spring and summer [91]. On the island of St. Kitts, 26% of 100 stray cats were infested with *C. felis* [92]. Fleas were the most common ectoparasite in homes in Costa Rica with 83% of dogs being infested with *C. felis* [93].

In South America, the most extensive surveys have been conducted in Brazil. Eighty-eight domestic dogs that lived outdoors were surveyed monthly for one year and only *C. felis* were collected. There was no significant correlation between temperature and infestation index and there was a negative association between infestation index and rainfall [94]. Paz et al. [94] concluded that seasonal differences in *C. felis* were likely due to climatic conditions in specific regions of Brazil. In a similar study, dogs from a farm in Brazil were surveyed for 1 year and two species of flea collected, *C. felis* and *P. irritans*. The number of cat fleas was significantly greater on long haired dogs than on short haired dogs [95]. Of 292 cats submitted to a spay/neuter program, 60% were infested with *C. felis* [96]. In rural northeastern Brazil, 18 of 29 dogs were infested with *C. felis* [97]. In two rural regions of Brazil, of the 328 dogs examined *C. felis* were found on 43.9 to 87.3% of the dogs depending upon the locality and season of the year [98]. In northeastern Brazil, 300 urban and 322 rural dogs were examined and 23.2% were infested with *C. felis*. More rural dogs were infested than urban dogs [99]. In Brazil, *C. felis* were the most common flea on dogs [100].

In other South American countries, 107 dogs from the domestic-wildlife interface in central Chile were surveyed and the following fleas were collected: *C. felis* (74.3%), *C. canis* (58.4%), *Pulex* sp. (11.8%) [101]. Studies in central Chile suggest that wild foxes (*Pseudalopex griseus*) and lesser grisons (*Galictus cuja*) could share fleas and potentially diseases with domesticated dogs. Fifty dogs were examined in Santiago, Concepción, and Osorno, Chile and of the 1000 fleas collected in each city *C. felis* represented 80.5, 38.4 and 6.6%, respectively. Osorno is more rural than the urban city of Santiago and this may explain the differences in species composition [102]. In Columbia, 140 dogs and 30 cats were surveyed and of the 3448 fleas collected 93.3% were *C. felis* [103]. *C. felis* was reported from French Guiana on cats and dogs [29].

*C. felis* is an opportunistic feeder and has been collected on wide range of feral hosts. A list of other hosts is provided in Table 1.

### 2.2. Biology and Life History

Adult *C. felis* exhibited a circadian rhythm with maximum activity occurring about 9 h into the light phase [104]. Mating never occurred off host. Fleas must feed continuously for maximum mating and insemination to occur. Sperm transfer and the insemination of females by males fed on blood from membranes was 84 and 45%, respectively. The juvenile hormone analogues (JHs), methoprene and pyriproxyfen, may indirectly regulate mating success by stimulating sperm transfer [105]. Males fed salt solutions or protein free diets did not inseminate females [106]. The exposure of fleas to the host’s body temperature and the amount of feeding were two factors that influenced insemination [107]. The mating behavior of *C. felis* has been described in detail [108,109]. Only fed males attempted to mate with unfed or fed females. The majority of mating occurred at 38 °C which is the body temperatures of cats and dogs. Interestingly, a chloroform:methanol extract from virgin females appears to contain a sex pheromone. Female *C. felis* mated with many males [109].

About 25% of fleas collected from the pelage of cats were engorged within 5 min and nearly all had fed within 1 h. The average duration of feeding was 25 and 10 min for female and male fleas, respectively [110]. Female fleas produced significantly more dry fecal droplets than did males. However, male and female adult fleas produced similar amounts of protein in their fecal droplets [111].

Significantly more fleas were collected on the head and neck of cats compared with the ventral body. The fewest fleas were collected on legs and tail [56]. Once adult fleas have established themselves on a host (48 h), the movement to an uninfected host was low (3.7%) [112]. About 33% of the original fleas were unaccounted for after 72 h. Greater numbers of fleas were removed by grooming from flea allergic cats compared with normal cats. Female fleas produced fewer eggs on cats with flea allergic dermatitis (FAD) than did fleas on normal cats even though feeding was the same. Some unknown factor may have reduced the number of eggs produced on FAD cats [113]. The effectiveness of grooming by cats to remove cat fleas varied from 4.1 to 17.6% of its flea burden daily. The mean longevity of fleas on the host was 7.8 days and females laid 38.4 eggs per day [114]. Once fleas attain the host, host grooming appears to be a significant mortality factor.

A multiplexed PCR assay has been developed that can determine blood meals of fleas from humans, cats, chickens, and rats. Humans and cats were the main blood sources for *C. f. strongylus* [115]. Another advancement using real-time PCR was the ability to detect human, rat and goat DNA in *C. felis* artificially fed up to 72 h post feeding [116]. Another novel PCR technique was developed that is sensitive and specific for blood ingested by cat fleas [117]. Increased gene expression during blood feeding in *C. felis* was investigated and revealed a number of genes activated during feeding. The proteins from these genes may be important in blood digestion, cellular activities and protection during feeding. This may open new avenues for control [118]. The salivary constituents, sialome, of *C. felis* includes many small polypeptides of unknown function. Parts of the sialome of *C. felis* are similar to that of *X. cheopis* [119].

Aspects of jumping behavior have been investigated, suggesting that both *C. canis* and *C. felis* are highly adapted for securing large moving hosts. *C. felis* has a faster jumping speed (average 3.6 m/s) than *X. cheopis* (average 1.4 m/s) [120]. The mean height of jumps for *C. canis* was 15.5 cm and 13.2 cm for *C. felis*, the highest jump being 25 and 17 cm for *C. canis* and *felis*, respectively. The mean length of jumps for *C. canis* and *felis* was 30.4 and 19.9 cm, respectively [121].

When flea infested cats were kept in a carpeted room, the flea eggs and larvae accumulated around the pet’s feeding and resting places [122]. Linardi et al. [123] reported that blood from 9 different mammals and birds was inadequate nutrition with only 33% of larvae pupating. Early instars depend upon essential dietary components and consequently spend more time in those food patches. Larvae spent the most time in patches with adult fecal blood and flea eggs [124]. Spray-dried bovine blood was a satisfactory lab diet for cat flea larvae [125]. Only 13.3% of larvae developed in to adults when fed flea feces compared with 90% when fed flea feces and non-viable flea eggs. However, larvae did not develop on flea eggs alone [126]. All of the *C. felis* larvae that fed on adult fecal material and frozen cat flea eggs developed into adults whereas only 6.6% that fed on fecal blood developed into adults. Larvae consumed >20 flea eggs in developing into adults. This may serve as a population regulating factor [127]. There is a direct positive relationship between yeast consumption and cocoon formation [128]. Only 3rd instars ate eggs whereas all instars ate yeast. Naked pupae were consumed by 3rd instar larvae whereas pupae inside cocoons were protected from predation. Substrates such as carpet afford larvae protection from cannibalism and increased their chances to successfully develop in to adults. In addition to specific nutrients, the relative humidity in the environment is critical for development. Larvae actively uptake water when the RH > 53%. Pre-pupae actively uptake water when the RH is between 75 and 93%. Pupae and adults do not actively up take water from the atmosphere [129].

Larvae survived outdoors in north-central Florida year round. From September to November survival was as high as 84.6%. In June and July eggs developed into adults in 20–24 days whereas in the winter it took 36–50 days. Immature stages survived frosts in protected microhabitats [130]. Male prepupae and pupae develop about 20% slower than do females [130,131]. At 15.5 °C, some adults emerge as late as 155 days after egg deposition [131], clearly showing the importance of the pupal and pre-emerged adult stage in surviving adverse conditions.

Over the years it has been of general interest to IPM practitioners to develop models that might predict the beginning of flea seasons. A meteorological model was developed to provide an index of weekly activity and an index of cumulative activity over 12 weeks. Only outdoor activity of fleas was considered in developing the model [132]. Keeping a dog indoors or cattle increased the prevalence of cat fleas captured on sticky cards on the floors of households in Yunnan Province, China and thus affected their model [133].

*C. felis* is known to have numerous endosymbionts, but their role remains largely unknown. In Australia, *C. felis* had less bacterial diversity than did a native *Echidnophaga* flea species [67]. Both species were dominated by the endosymbiont *Wolbachia*. *Wolbachia* vary among different species of fleas and the practical implications are unknown [134]. An intestinal gregarine, *Steinima ctenocephali*, neither affected the emergence rates or survival of fleas. Flea larvae with the gregarine developed faster than those without them [135]. A tryponsomatid *Leptomonas ctenocephali* was found in the digestive tract of adult cat fleas, but its capacity to be pathogenic to fleas or the hosts has yet to be determined [136].

## 3. Veterinary and Medical Importance

More than US$15 billion are spent on protecting companion animals from fleas annually [137]. Parasiticides represent 46% of the companion health market worldwide with ectoparasiticides being the largest segment [138]. In the US, about 65% of all households have companion animals, mostly cats and dogs, and fleas and ticks represent a considerable concern and investment by the public in the welfare of their pets. In 2011, it is estimated that US$2.4 billion was spent on anti-parasiticides for small animals. This market continues to grow at a compound annual growth of 5% [139]. However, the costs of developing new parasticides are enormous and this certainly will be a factor in the continuing efforts to develop and register parasiticides for small animals [140].

A random sample of 3584 cats from a 142,576 cat data base from 2009–2014 in the UK revealed that flea infestations were the second most common disorder of cats (8.0% prevalence) [141]. In Portugal, 312 pet owners that visited the animal hospital at the University of Lisbon were surveyed and 81% used ectoparasiticides resulting in 92.2% of dogs and 52.7% of cats being treated [142]. In Hungary, only 49% and 37.6% of dog and cat owners, respectively, knew that their animals were infested with fleas [69]. An examination of 5276 dogs and 1226 cats revealed that 50–80% of dogs and 38% of cats were on preventative flea and tick products. In April–May, 80% of dogs were on preventatives, but that fell to 50% from October to December [143]. In a US survey of 24 veterinary hospitals, it was estimated that dogs were given preventative treatments about 6.1 months each year based on medication purchases. Even though the staff at hospitals recommended protection for 12 months, only 62% of the dog owners remembered that recommendation [144]. In Portugal, a survey of pet owners revealed a lack of general knowledge of zoonotic diseases and their vectors. About 50% of the dogs were treated monthly, but 42% of the pet owners frequently forgot to re-treat the pet [142]. Clearly, better education of the benefits of preventative care for the pet owner is needed.

The public health and veterinary significance of fleas have been reviewed on a number of occasions [3,4,5,6,7,145,146,147,148]. However, the prevalence of flea-borne diseases has been greatly underestimated by health practitioners and agencies [149,150]. With the development of new molecular research tools the prevalence and potential importance of rickettsial diseases such as murine typhus and flea-borne spotted fever and bartonelloses in companion animals and humans are being elucidated. For example, in a study of 121 dogs and cats in the UK, 50% of samples were PCR positive for at least one pathogen including 21% with *Rickettsia felis*, 17% with *Bartonella henselae*, and 40% for haemoplasma species [151].

### 3.1. Rickettsial Diseases

In recent years, there have been a number of reviews [149,152,153,154,155,156,157,158] and scores of papers primarly dealing with rickettsial diseases of cats and humans and simply too many to include in this review. Two rickettsial species, *Rickettsia felis* and *R. typhi*, are transmitted to humans through flea vectors. *R. felis* is an intracellular Gram negative bacterium and an emerging pathogen worldwide, but its ecology and epidemiology are still not completely understood [154,155,159]. Much of the initial research was focused in sub-Saharan Africa because of its prevalence there [151], but now is literally being studied globally.

Opossums and domestic animals appear to be involved with the *R. typhi* life cycle, the causal agent of murine typhus, in Austin, Texas [160]. Of the animals tested, 18%, 24% and 71% of the cats, dogs and opossums, respectively, were seropositive for *R. typhi* antigen. All of the opossums (18) and 12/17 of the cats were infested with *C. felis*. Molecular evidence of *R. typhi* was found in 7 of 12 opossums [49]. Overall, rickettsial infections were detected in 37.2% of the cat fleas analyzed from opossums and cats [161]. Billeter and Metzger write, “In California, field studies focused on cat fleas as possible vectors of *R. felis* do not corroborate or explain the incidence or distribution of human disease. Instead, these studies raise doubts regarding the significance of these fleas in the epidemiology of flea-borne rickettsioses” [159]. Further studies are clearly warranted to establish the relationship of murine typhus and cat fleas.

### 3.2. Bartonellosis

The *Bartonella* species are small intracellular Gram-negative bacteria that are vector borne. There some 22 different species of *Bartonella*, but *Bartonella henselae* is the species most commonly found in humans and cats [162]. In recent years there have been a considerable number of papers and reviews that focus on the disease and too many to include in this review [156,158,162,163,164,165,166]. Several species of the Gram-negative bacteria in the genus *Bartonella* have been reported in fleas. However, the role of fleas as vectors has not been adequately studied [149]. *C. felis* is the main vector of *B. henselae* or cat scratch disease (CSD) with the bacteria being inoculated into the host from contaminated fecal material in to a cut or scratch [153]. Dogs can be infected with various species of *Bartonella*, but their role as a reservoir is not well understood [166]. In New Zealand 18.9% of the fleas contained DNA from *B. clarridgeiae* suggesting that dogs and cats may serve as a reservoir [20].

The occurrence of human CSD is not reported nationwide and it is unclear what the prevalence of this disease is among the population [153]. However, it is commonly diagnosed in children [167]. Most cases in humans are self-limited and do not require antibiotics. McElroy et al. write, “Currently, because of the difficulty in identifying animals that would benefit from therapy, antimicrobial agents are not recommended to treat or prevent *Bartonella* infections in cats” [153]. No vaccines are yet available and flea and tick control seem to be the best preventive option [162]. Even though the direct treatment of cats and dogs for rickettsial diseases may not be a primary recommendation, minimizing the risk of transmission by providing flea control is clearly important [3]. Strict flea control is the only successful preventive measure [162] and control of fleas on cats and other prevention strategies are helpful in households with children [164].

### 3.3. Plague

Laboratory studies indicate that *C. felis* is a competent vector of *Yersinia pestis;* however, the efficiency is low compared with vectors such as *Xenopyslla cheopis* [156,168]. In fact, the rapid turnover of midgut contents in *C. felis* and the disruption of the biofilm accumulation in the proventriculus greatly reduce the likelihood of *C. felis* being a potential vector of plague [169].

In some plague-infested regions of Uganda, *C. felis* is the most common flea in dwellings and occasionally infests rodent reservoirs of plague. Clearly rat flea populations need to be reduced in plague control programs, but the control of cat fleas should not be ignored because of their potential as secondary vectors [168].

### 3.4. Tapeworms

*C. felis* is the intermediate host of the cestode *Dipylidium caninum*. Larval cat fleas become infected by consuming the cestode egg and the infective cysticercoid develops in the adult flea. Mammalian host become infected with the tapeworm when infected adult fleas are consumed. Utilizing a PCR test, *D. caninum* rDNA was detected in individual fleas collected from nine European countries. Of the 4365 cats surveyed 4.4% were infested with *C. felis* infected with *D. caninum* [170]. Of the 1500 *C. felis* collected from 1590 dogs in Brazil, 0.4% had *D. caninum* [171]. In Mexico, 36% of the 358 cats had *D. caninum*. Nearly 75% of 358 cats were stray cats and probably explains the high rate of infestation [172]. In Romania, 0.2% of the cats examined had *D. caninum* [173]. In western Hungary, taenid cestodes were found in 1.7% of the cat fecal samples examined [70]. These recent studies provide a clearer picture of tapeworm prevalence in pet populations.

### 3.5. Flea Allergic Dermatitis (FAD)

Sensitivity to flea bites and the development of flea allergic dermatitis are common to both cats and dogs [3,8,174,175,176]. There is indirect evidence to support a hypothesis in canines that atopy predisposes to the development of hypersensitivity to flea allergens and eventually to FAD [177]. Occasionally severe puritanism and inflammation is reported in humans to flea bites [178]. There was one case report of a flea infestation inside a hospital with humans being bit [179]. Six students from Malaysia reported to a hospital with bites from *C. felis* [180].

A survey of 163 dogs and cats revealed that 58.3% had symptoms of FAD with dogs >4 years old and cats from 1 to 4 years old being the most affected. Companion animals <1 year old were less susceptible to FAD [103]. In a large study in the UK, 1.9% of the cats examined were hypersensitive to flea bites [142].

Antibodies in the sera of mice implied that 4 potential antigens in the salivary glands of fleas may be responsible for the flea bite hypersensitivity [181]. Intradermal tests with dogs indicate that proteins MW 40 k and MW 12 k–18 k were important in flea bite sensitivity [182]. A major flea salivary allergen (*Ctef1*) was identified and cloned [183]. Platelet-activating factor–acetylhydrolase activity has been demonstrated in cat flea saliva suggesting that this might limit local inflammation and immune responses by the host [184].

### 3.6. Other Diseases and Pathogens

Laboratory studies were conducted to determine if *C. felis* was a potential vector for feline leukemia virus (FLV). FLV is ingested and viral RNA was excreted by adult fleas and remained in the adult fleas for up to 30 h. Half of the original amount of FLV ingested remained in the feces for two weeks. Viral RNA was also directly transmitted during feeding. The clinical significance of this remains unknown [185,186,187].

Cat fleas collected from foxes and rats were positive for *Coxiella burnetii* by PCR tests [188]. *C. burnetii* is a gram negative bacteria that infects a number of domestic and feral animals, primarily ungulates. The role of fleas in is maintenance and transmission has yet to be determined.

The flea feces collected from fleas that fed on blood spiked with feline calicivirus thorough membranes contained active virus. Kittens were infected by the flea feces and in one case from the feeding of an adult flea [189].

The cat flea is considered the main vector of the helminth worm, *Acanthocheilonema reconditium*, even though it is unknown if the infective larvae are transmitted via the flea bite or when consumed [190].

The ability to transfer leishmaniasis by *C. felis* was tested, but the possibility of oral transmission could not be shown unambiguously [191].

## 4. Rearing and Testing Methodologies

The maintenance of *C. felis* colonies on cats or dogs is laborious and expensive. The use of large numbers of animals to rear and test potential flea products is also a concern of animal rights activists. Thus, artificial membrane testing and alternative rearing procedures are of special interest and the expanded use of these techniques could drastically reduce the need and costs for large numbers of laboratory animals. Various mammalian bloods, including bovine, ovine, porcine, and human, have been tested with artificial membrane feeding systems with varying degrees of success [192]. The use of EDTA as an anticoagulant resulted in increased numbers of flea eggs, but the percentage of eggs developing to adults was low. The highest egg production occurred when 25 ♂♂ and 100 ♀♀ were held together [192]. Considerably more research needs to be conducted with various laboratory strains and field-collected isolates of *C. felis* to better understand the limits and potential problems associated with membrane-fed flea populations. This research could provide tremendous cost savings and the need for laboratory animals.

*C. felis* maintained on rats consumed more blood, produced more eggs and had higher sex ratios of offspring than did those that were fed on mice. It is unclear if the lower numbers of fleas obtained were due to increased grooming by the mice [193]. A mass rearing method of *C. felis* was developed on mice in which *C. felis* females laid an average of 10.3 eggs/day which is considerably lower than female fleas maintained on cats. Adult *C. felis* survived for >40 days on the mice [194]. Another advantage is that sedated mice can be dosed with systemic insecticides and tested. Mice were dosed with active ingredients such as nitenpyram, cythioate, and fipronil and adult *C. felis* allowed to feed on them. Nitenpyram (1 mg/kg), cythioate (10 mg/kg), and fipronil (30 mg/kg) provided >94%, 64%, and 83% mortality, respectively. The mice might serve as a test model, possibly reducing the numbers of larger animals such as cats and dogs for systemic testing [195].

The WHO bioassay of exposing adult fleas to treated filter paper strips treated with insecticides has been the standard procedure used to detect insecticide resistance in fleas [196]. Franc and Cadiergues [197] reported the LD_50_’s of deltamethrin, permethrin, bioallethrin, and esbiothrin were 0.38, 230, 121, and 161 mg/m^2^, respectively. In a modified WHO test, the contact activity of insecticides applied to glass and nylon fabric substrates was compared with filter paper strips in adult flea exposures [198]. The nature of the substrate greatly affected the toxicity of insecticides such as carbaryl, malathion, permethrin and pyrethrum. Prior exposure of adult fleas to CO_2_ increased their susceptibility to insecticides, but circadian rhythms had no effect on toxicity [199].

The intrinsic activity of 13 different insecticides was tested against adult fleas by means of topical applications [200]. The test provided precise doses required to kill fleas, but requires considerable numbers of adult fleas and the laboratory maintenance of field-collected strains. A bioassay was developed to screen the potential activity of compounds against individual fleas in 96-well tissue culture plates. The bioassay distinguished between contact toxicity and insect growth regulator (IGR) effects [201]. Similarly, Chen et al. reported a contact and oral bioassay to test individual larva also using 96-well microliter plates [202]. In this bioassay, the laboratory strain was 2–4 times more susceptible to fipronil than the field isolate of *C. felis* tested, but there was no difference in susceptibility with imidacloprid or spinosad.

A larval bioassay was developed to determine the susceptibility of *C. felis* to imidacloprid utilizing flea eggs. The collection and shipment of flea eggs allowed the research team to collect field isolates from numerous clinics throughout 7 countries. Flea eggs were suspended over larval rearing medium and allowed to hatch thereby reducing the cannibalism of flea eggs [203]. To expedite the testing of large numbers of field-collected isolates a diagnostic dose of imidacloprid was determined to be 3 ppm [204]. This dose was robust enough to eliminate most isolates, but low enough to identify potential isolates for additional resistance testing. Topical applications of imidacloprid and fipronil to adults and exposure of larvae to treated media provided similar results for field-collected isolates and laboratory strains verifying the utility of the larval bioassay [205].

An improved bioassay technique was developed with treated filter paper strips to determine repellency of compounds to adult fleas. Deposits of 2% trans-cinnamaldehyde and 0.5% thymol repelled 97.6 and 90.6% of fleas for at least 8 h which was comparable to 15% DEET [206].

## 5. Pest Management

The prevention and control of cat fleas have been the subject of many reviews and commentaries [3,4,6,8,9,10,11,207,208,209,210]. There have been a number of reviews of the new active ingredients and products used to control *C. felis* since 1997 [3,145,146,210,211,212,213,214,215,216,217,218]. Pfister and Armstrong provide a review and comparison of the systemic fluralaner and the cutaneous permethrin against fleas and ticks [219]. Woodward provides a review of insecticides in veterinary products that focuses on their toxicology [220].

Testing with active ingredients available in the marketplace in 1997 continues and many new active ingredients have been registered. In addition, products consisting of several active ingredients have also been registered. In the past 20 years numerous new active ingredients have also appeared. A standard for performance and overall efficacy at the end of the time period as established by the European Medicine Agency is 95% kill of fleas [221] and in the US, EPA accepts 90% kill as a standard. The need for more universal standards worldwide has been addressed by Bobey [138]. Based on counts in the control and treated groups, the speed of kill occurs when at least 95% of the fleas are killed in both the control and treated groups. These standards are consistent with guidelines of the World Association for the Advancement of Veterinary Parasitology will be considered when reporting the following review of efficacy studies [222,223]. Positive controls (a standard reference product) are recommended to validate on-animal treatments and thus many studies report comparative efficacy data to other existing products at the time of the study. Factors that may contribute to apparent variation in laboratory data include the strain of cat flea being tested, substrates being treated, exposure periods, and the duration of the tests. Caution is advised when reviewing and comparing the following studies.

Laboratory and field studies with active ingredients such as fipronil, imidacloprid, lufenuron, methoprene, permethrin, and pyriproxyfen registered prior to the 1997 have continued. Changes in formulations, application technology, the combination with other active ingredients, and generic products have been reported for many of the older active ingredients. For example, a formulation of permethrin spot-on that contained propylene glycol monomethyl ether provided 93–99% kill of fleas on dogs from day 3 to day 28 where as the original registered formulation containing diethylene glycol monomethyl ether provided only 48% at day 28 [224]. In another study, both formulations provided >95% kill of adult fleas for at least 28 days [225]. Cats treated with an experimental formulation of fipronil spot-on formulation containing dimethyl sulfoxide provided >95% kill for up to 5 weeks [226] and similar test were conducted on dogs [227].

Deltamethrin shampoo provided 100% efficacy adult *C. felis* at 24 h and >95% protection for at least 17 days [228]. Deltamethrin shampoo on dogs prevented >98% flea feeding for at least 3 days, but by day 14 the protection against feeding had declined to 30.1% [229].

Sprays containing 0.29% fipronil applied to cats provided >99% reduction with a susceptible laboratory strain of cat fleas, but provided only 77.3% adult kill and 87.3% egg reduction at day 30 when tested against a field-collected isolate [230]. Topical applications of fipronil/methoprene, imidacloprid/permethrin, or imidacloprid to dogs provided 96, 48 and 74% kill, respectively, at day 28 when fleas were counted at 24 h [231]. Topical applications of fipronil/methoprene to cats provided >95% kill for 28 days when counted at 24 h. Egg production was reduced by 77–96% for 42 days, and none of the eggs collected developed for at least 56 days [232]. A generic formulation of fipronil provided up to 8 weeks efficacy against *C. felis* on dogs [233] and up to 6 weeks on cats [234]. Another generic formulation of fipronil/methoprene applied to infested dogs provided 38% kill of fleas by day 3. Mortality increased to 95% by day 21 and 100% by day 28 [235]. A topical treatment of 10% fipronil on dogs provided >95% kill for 35 days when challenged with either 100 or 300 unfed fleas. At day 42, the efficacy declined to about 68% in both challenges [236]. A spot-on combination of fipronil/methoprene on dogs provided >95% kill of adult fleas for 5 weeks. The fipronil/methoprene combination provided >90% ovicidal activity and 91% inhibition of adult emergence for 8 weeks and the authors propose that the combination may be synergistic against the immature stages of fleas [237].

Imidacloprid applied to cats and dogs provided >95% kill of adult fleas for at least 3 weeks when fleas were counted at 24 h and >95% for at least 4 weeks when counted at 48 h [238,239]. The synergist piperonyl butoxide significantly increased the activity of technical imidacloprid against adult fleas at 26 °C, but with mixed affects at 20, 30, and 35 °C [239]. Richman et al. suggested that interference with several detoxification mechanisms may occur increasing the activity of imidacloprid [240]. The combination product imidacloprid/moxidectin provided >98% control of adult fleas for at least 28 days [241]. Imidacloprid/moxidectin provided significant reductions in adult fleas and prevented the transmission of *B. henselae* to cats [242]. In a comparative study of commercial products, topical application to dogs of imidacloprid provided >95% kill of fleas on dogs for at least 37 days. Diazinon, permethrin, and fipronil provided >95% kill for at least 2 days [243]. In simulated home environments, spot-on applications of imidacloprid and fipronil provided nearly complete control of fleas. Lufenuron required an additional treatment and mechanical removal of adult fleas to achieve control [244].

A topical application of permethrin/pyriproxyfen to dogs provided 90–100% kill of fleas for up to 3 weeks and 100% ovicidal effect for 49 days [245]. Permethrin/pyriproxyfen sprays applied to dogs provided >90% knockdown of fleas within 15 min and prevented more than 94% of them from feeding for up to 2 weeks. Sprays containing fipronil and imidacloprid provided significantly less knockdown and antifeeding activity compared with permethrin sprays 4 h after treatment [246]. Sprays containing synergized *d*-allethrin/pyriproxyfen applied to cats in simulated home environments resulted in a gradual reduction of adult fleas on the cats and a 100% reduction of eggs, larvae, and adults within 41, 19, and 23 days, respectively [247].

The combination of IGRs and adulticides or the use of IGRs alone continues to be interest [124]. IGRs affect eggs, larvae and adult fleas and the combination with adulticides applied to cats and dogs has been shown to prevent cat flea eggs from hatching and larval from developing in the environment. In addition, IGRs appear to decrease the time required to control fleas indoors and may also lessen the likelihood that insecticide resistance will develop [3].

Lufenuron mixed into blood at 1 ppm and fed to adult fleas through a membrane prevented 98% of flea eggs from hatching. The abnormal formation of the procuticle of lufenuron treated larvae resulted in their death at eclosion [248]. An injectable formulation of lufenuron in cats provided >95% control by week 9 and this continued at >90% reductions for 26 weeks in simulated home environments [249]. Similarly, injectable formulations of 10 and 20 mg/kg lufenuron in cats resulted in 90% reduction of eggs developing into adults for 196 days [250]. In a clinical study, dogs and cats were dosed monthly with lufenuron for 3 years. None of the treated pets were infested with fleas at the end of the study. All of the homes and pets in the controls were infested with fleas at the end of the study [251]. A year-long field study in Cairns, Australia found that nitenpyram and lufenuron provided 90–100% reduction of fleas on the pets and in the house. The results with imidacloprid were variable with an initial 84% reduction during the first 16 weeks that dipped to 18%, and then returned to 70–84% for remainder of study [252].

The formation of the chorion of the flea has been described in detail providing background for examining the effects of IGRs [253]. Lufenuron disrupted the formation of endocuticle in the larvae and caused degeneration of the epidermal cells [254]. Blood containing 2–4 ppm lufenuron fed to adult fleas resulted in 18–24% mortality at day 10 [255]. Lufenuron caused degeneration of epidermal cells and inhibition of midgut epithelial cell differentiation.

Blood containing pyriproxyfen fed to adult fleas through a membrane was relatively non-toxic to them. However, the eggs were not viable and failed to hatch [256]. Similarly, 100% of eggs collected from cats treated with pyriproxyfen after treatment failed to hatch. Excellent residual activity persisted for at least 60 days [257]. In a large field trial, 107 flea-infested cats were treated with a pyriproxyfen spot-on formulation and 99 cats treated given lufenuron once a month. On day 30, 49% of the pyriproxyfen treated cats were flea-free and this increased to 88% by day 180. Of the cats dosed with lufenuron, 30% and 71% of them were flea free at day 30 and 180, respectively [258]. Exposure of eggs and larvae to hair treated with 0.01 μg/kg AI completely inhibited development. When adult fleas were exposed to pyriproxyfen for 3 days, the eggs collected for the next 14 days did not develop. Exposure for just 2 h provided 100% inhibition [259].

Carpet exposure and larval media studies showed that pyriproxyfen residues were more active than methoprene or hydroprene [260]. Methoprene plus permethrin increased the kill of pupae in certain carpets [261]. All stages were tolerant to residues of the IGRs, methoprene and pyriproxyfen, on glass. When exposed to treated surfaces, larvae were unable to pupate. Pharate pupae ecdysed into pupae, but could not eclose. Pupae and adults were unaffected [262]. The LD_50_ of methoprene and pyriproxyfen applied to carpet after aging 12 months was 0.2–1.0 and 0.04–0.2 mg/m^2^, respectively [263].

Debris collected from cats treated with imidacloprid provided >95% kill of larvae for at least 61 days after treatment [239]. Blankets in contact with cats treated with imidacloprid prevented 100% and 74% of larvae from developing in to adults for 1 and 4 weeks, respectively [264,265].

Samples of hair from dogs and cats treated with pyriproxyfen were analyzed for pyriproxyfen. Initial samples contained 0.2 to 4.16 mg/kg on dogs and cats, respectively. At 8 weeks the levels still exceeded 0.02 to 0.21 mg/kg on dogs and cats, respectively. Only 0.0001 mg/kg pyriproxyfen is necessary to provide excellent control of flea larvae [266]. Flea eggs collected from cats treated with topical pyriproxyfen failed to hatch for up to 7 weeks. Flea larvae in contact with blankets from cages with treated cats failed to develop into adult fleas and the residual activity persisted for at least 2 weeks [267]. Eggs collected from dogs treated monthly with lufenuron-milbemycin failed to hatch for the day test period [268].

Pyriproxyfen synergized methoprene with as little as 0.06 ppm treated larval media prevented adult emergence by 50% [269]. Other IGRs including chlorfluazuron, cyromazine, dicyclanil, and precocene were active against *C. felis* larvae with chlorfluazuron and dicyclanil being more active than methoprene or pyriproxyfen [270]. When eggs and larvae of *C. felis* exposed to filter papers treated with pyriproxyfen, adult emergence was inhibited at 0.1 μg/m^2^ [271]. CGA-255’728 mimicked the effect of JH, especially at rates >100 ppb, but this compound appears not to have been developed against cat fleas [272].

When pupae were treated with methoprene or pyriproxyfen, there was a significant increase in adult mortality within 48 h [273]. Adult mortality was 45.8% with methoprene, 48.4% with pyriproxyfen and only 1.3 to 4.3% in controls. No effect was observed on the fecundity of surviving fleas. IGRs have multiple effects on immature and adult fleas increasing the efficacy of combination treatments.

### 5.1. New Active Ingredients

New active ingredients and combination treatments continue to be investigated and registered as on-animal and oral therapies even though there are a number of excellent products already in the marketplace. Their development appears to be driven by marketing issues such as convenience, safety, cost, and the need for treatments that control a variety of arthropod pests. However, increased convenience for the consumer can lead to over-use and drug resistance [142]. In an effort to broaden the biological activity of products, numerous combinations of insecticides have been tested and registered in the past 2 decades [210]. Four basic types of efficacy studies are typically reported in the literature: (a) laboratory in vitro; (b) on-animal studies in the laboratory; (c) on-animal studies in simulated home environments; and (d) clinical field studies. Table 2 provides a summary of tests conducted with new active ingredients and combination products registered for cat flea control since 1997.

#### 5.1.1. Laboratory In Vitro Studies

Initial bioassays often include continuous exposure tests with adult or larval cat fleas on treated surfaces. Several compounds from a series of 2-phenyl-3-(1*H*-pyrrol-2-yl) acrylonitrile derivatives exhibited contact toxicity against adult *C. felis* [370]. Topical applications to adult cat fleas of a number of 2-alkoxy- and 2-aryl-oxyimino-aryl trifluoromethansulfoonanilides were very active [371]. An extract of neem seeds showed contact activity against both larvae and adult *C. felis* [372]. Deposits of A1443 (fluralaner) on naglene petri dishes had slightly less contact toxicity than did fipronil deposits to adult fleas [373].

Hair clipped from animals treated with commercial products containing imidacloprid, fipronil, and selamectin killed adult fleas with 1.5, 29, and 96 h, respectively. Unfortunately, high larval mortality in the controls prevented any analysis of the activity against the larvae [364]. Larval rearing media treated with solutions of technical selamectin provided greater kill than did technical imidacloprid or fipronil with as little as 1.3 ppm selamectin providing 100% kill when fleas were counted at 72 h [274].

Membrane feeding studies with *C. felis* have been used to explore the insecticidal activity of nodulisporic acid and analogs [374,375]. The LC_50_ for nodulisporic acid for adult fleas feeding through a membrane was 0.68 μg/mL [374]. A series of nodulisporamide compounds were evaluated for systematic activity against *C. felis* on cats and dogs and *N*-tert-butyl nodulisporamide showed best results [376]. Utilizing a membrane feeding system, avermectin derivatives were screened discovering the compound selamectin [275]. An oral dose of 5–10 mg/kg or 15–20 mg/kg selamectin per dog or cat, respectively, provided 100% kill of fleas for 30 days [376]. Ivermectin administered through feeding membranes had a LC_50_ and LC_95_ of 19.1 and 9.9 μg/mL, respectively, but the primary conclusion was that even the best avermectin did not have the potential as a commercial oral or subcutaneous flea treatment [377]. In membrane feeding studies, afoxolaner was shown to be highly active against adult *C. felis* with as little as 0.16 μg/mL killing 100% within 24 h [378]. Membrane feeding studies with fluralaner were conducted against a cat flea strain that had *cfrdl*-S_285_ genotype expected to confer dieldrin resistance and as little as 0.1 ppm killed adults, an order of magnitude more active than fipronil or imidacloprid [379]. Doses as low as 12.5 ng/mL fluralaner provided 100% disruption of flea reproduction in membrane feeding tests [365]. In vitro studies showed that sarolaner was about 10 times more active than afoxolaner or fluralaner against adult fleas [366].

Radio-labelled studies have been conducted to determine how insecticides move on the host and within the fleas’ body. Radio-labelled fipronil applied to the skin of the dog was found in the strateum coneum, viable epidermis, the sebaceous glands and epithelial layers for up to 56 days [360]. Using radio-labelled selamectin and ivermectin, there were increased quantities of selamectin in the flea ganglia at glutamate-gated chloride channels and it may in part explain its increased toxicity over ivermectin [276].

Electrophysiological recordings of isolated neuronal cell bodies and in vitro bioassays against *C. felis* adults suggest that the combination of imidacloprid and flumethrin were synergistic [353]. A combination of dinotefuran/fipronil showed strong synergism against adult fleas in exposure studies on glass deposits [380]. The interaction of multiple insecticides when mixed in combinations certainly warrants additional research.

#### 5.1.2. On-Animal Studies in the Laboratory

The majority of efficacy tests of potential insecticides to control fleas is conducted on cats and dogs confined indoors eliminating environmental factors and external sources of fleas. In such studies, the kill of 90–95% of the adult fleas is considered the benchmark by different regulatory agencies and the time at which the fleas are counted varies between 2 to 72 h depending upon the study [222,223]. In vitro bioassays may not always directly correlate with in vivo studies. Many studies include other registered compounds for comparative purposes. Many of the slower acting insecticides have performed very well in simulated field studies and clinical studies. The studies have been presented in chronological order to provide a history of the testing and development of new products as they occurred.

Sprays containing 2400 ppm azadirachtin or higher applied to dogs provided >95% kill of adult fleas for at least 5 days [381]. There was a 99% reduction in *C. felis* on dogs provided an oral dose of nodulisporic acid at 15 mg/kg when fleas were counted at 48 h [374]. Neither have been developed as control products.

A topical application of 6 mg/kg of selamectin for cats and dogs was determined to be the most effective dose, killing >95% of fleas when tested at day 30 [278]. Of the eggs collected from treated animals 92% failed to hatch and 85–100% of the larvae failed to develop into adults. Debris from treated animals also prevented egg hatch and larval development [279]. A minimum dose of 6 mg/kg of selamectin provided >97% reductions of fleas even after shampooing both cats and dogs [280]. Topical doses of selamectin, imidacloprid, and fipronil provided >95% kill of fleas for 29 days on dogs when fleas were counted at 72 h. Repeated monthly applications provided >95% kill for the next 120 days [281]. In a similar study on cats, monthly applications of fipronil and selamectin provided >98% reductions of fleas for 120 days [282]. Similarly, topical applications (4 or 8 mg/kg) or oral doses (2 mg/kg) of selamectin provided >95% reductions of fleas on cats and dogs when counted after 48 h on day 30 [277]. Spot-on applications of selamectin, imidacloprid and fipronil to dogs provided 100% kill of fleas for at least 30 days [283]. Topical application of selamectin to pregnant and lactating female dogs provided >99% kill of fleas on the mothers and 100% kill of fleas on the pups [284]. Selamectin, imidacloprid and fipronil applied to cats provided >95% kill when counted at 48 h for at least 37 days [285]. Topical selamectin, oral spinosad/milbemycin oxime, and oral spinosad killed >90% of fleas on dogs within 24 h when tested at days 7, 14, and 21. Selamectin provided >93% reductions at day 28 whereas two spinosad products provided <90% kill [286]. A monthly topical application of selamectin was applied to dogs and cats with FAD. Selamectin provided >95% control of fleas throughout the 84 day study. Signs of FAD dramatically improved on the dogs and somewhat less with the cats [287].

Adult fleas were knocked down within 30 min after providing an oral dose of nitenpyram to cats or dogs [299]. Within 6 h after administration, 96.7% and 95.2% of fleas on dogs and cats, respectively, were killed. Nitenpyram provided 100% kill of adult fleas from a field-collected strain resistant to fipronil within 24 h [300]. Doses of nitenpyram to cats provided 100% kill of fleas at the time of treatment and for up to 24 h. Between 24 and 48 h 98.6% of adult fleas were killed and after 72 h efficacy declined to 5%. Egg production decreased by more than 95% in the first 72 h after treatment [382]. Within 4 h, oral doses of nitenpyram killed 100% of *C. felis* on dogs, whereas topical applications of fipronil and imidacloprid killed 36.9% and 78.4% of the fleas, respectively [298].

A combination of imidacloprid/permethrin topically applied to dogs provided >95% kill of fleas for 4 weeks. Blankets held within cages of treated dogs provided >85% inhibition of larval development for 4 weeks [334]. A combination of fipronil/permethrin spot-on treatment on dogs provided >98.4% kill of adult fleas for 28 days. Neither shampooing or immersing the dogs in water 2 or 3 times affected the efficacy [352].

Repeated monthly doses of 30 mg/kg spinosad to dogs provided >95% kill of fleas at day 90 [341]. When counted 12 h after challenging with fleas, spinosad provided >95% kill for at least 21 days. An oral dose of spinosad to dogs resulted in 100% adult flea kill at 4 h and >99.5% reduction in flea egg production over 30 days. There was no toxic effect from debris collected from beneath the treated dogs. Egg hatch of eggs collected from dogs treated monthly with spinosad varied providing inconsistent results [342]. Topical treatment of fipronil/methoprene to dogs provided 100% kill through 6 weeks. By day 28, the efficacy of spinosad fell to 89% when fleas were counted at 48 h. No eggs were collected from fipronil/methoprene group whereas at day 37 more than 100 eggs were collected from each dog in spinosad group [302]. An oral dose of spinosad provided >95% kill for 30 days on dogs [285]. An oral dose of spinosad against *C. felis* on dogs provided >95% kill of fleas for 22 days compared with 100% kill with fipronil/methoprene at day 43 [295]. A combination spinosad/milbemycin oxime and spinosad applied to dogs provided 100% kill of fleas for at least 30 days [350].

A topical application of 20 mg/kg or 40 mg/kg metaflumizone/amitraz to dogs provided >95% kill of fleas for at least 35 days [338]. A single spot on application of metaflumizone on cats provided >90% kill of fleas for up to 7 weeks [383]. In another study, metaflumizone applied to cats (40 mg/kg) provided 97.4% kill of fleas for 4 weeks compared with 71.3% kill for fipronil/methoprene [340].

Topical applications of pyriprole to dogs provided >95% kill for 35 days and similar results for animals washed once each week [383]. A topical application of pyriprole on dogs prevented fleas from laying eggs for at least 30 days [384].

A topical application of dinotefuran/pyriproxyfen to cats provided a 83.9% reduction in the number of fleas counted at 2 h and a 100% reduction at 6 h. Its residual activity provided >99% kill at day 30 [385]. Dinotefuran/pyriproxyfen/permethrin and dinotefuran/pyriproxyfen applied topically to dogs killed >99.5% of fleas for at least 28 days whereas an oral dose of spinosad provided 22–38% kill [328]. A spot-on application of dinotefuran/pyriproxyfen to dogs provided 100% kill within 24 h and >96% reduction of fleas for at least 56 days [386]. A topical application of dinotefuran/pyriproxyfen/permethrin provided rapid kill of fleas >95% at 6 h and >96.8% kill at 1 month [343]. A single treatment of dinotefuran/pyriproxyfen/permethrin provided 99.7% kill of adult fleas and 96.2% kill at day 30 [347]. Egg laying inhibition was >92.3% for up to 29 days and there was 100% inhibition of adult emergence for 8 weeks after treatment.

The efficacy of a topical application of an experimental formulation of cyphenothrin/pyriproxyfen on dogs provided >97% kill of *C. felis* for at least 30 days after treatment. The efficacy declined to 56.8% at day 44 [387].

Indoxacarb topically applied to cats provided 99.6% kill of adult fleas at day 42 and reduced flea egg production by 95.5% at day 45 [348]. Spot-on applications of indoxacarb on dogs that were shampooed provided >95% kill of adult fleas for at least 30 days [349].

A single application fipronil/methoprene or 2 successive applications of fipronil/methoprene/amitraz provided >97% efficacy on both cats and dogs. Both treatments prevented the establishment of *Dipylidium caninum*. Dogs treated monthly with fipronil/methoprene and fipronil/methoprene/amitraz provided >97.5% efficacy when fleas were counted at 24 h from day 135 to 232 [303].

Initial studies with afoxolaner indicated that an oral dose of 25 mg/kg to dogs provided >98% flea reduction at day 32 [304]. In a later study, an oral dose of afoxolaner (2.5 mg/kg) to dogs killed >98.8% of adult fleas for 32 days [378]. Similarly, afoxolaner given to dogs provided >95% kill of *C. felis* for 21 days when fleas were counted at 12 h and 100% kill for 35 days when counted at 24 h [305]. Flea egg production was reduced by more than 99% for 35 days. In another study, oral doses of afoxolaner to dogs provided consistently higher mortality of fleas than did fluralaner at 6 h. Afoxolaner and fluralaner killed 100% of fleas when counted at 24 h for at least 77 days [306].

Oral doses of sarolaner ranging from 1.25 to 5.0 mg/kg for dogs provided >99% kill of fleas when counted at 24 h for at least 35 days [307,366]. An oral dose of sarolaner (2.0 mg/kg) provided >99% kill of laboratory and field-collected strains of cat fleas when counted at 24 h for at least 35 days [308]. Similarly, a single dose of sarolaner killed >95% of fleas for at least 35 days when counted at 24 h. There were no flea eggs collected from dogs dosed with sarolaner [309]. A topical treatment of an experimental selamectin/sarolaner combination to cats provided 100% efficacy for up to 36 days. A few eggs were collected, but they failed to develop [310].

A single oral dose of fluralaner provided 100% kill of adult fleas for 4 months on dogs [311]. Initially, egg production was reduced by 99.9% and in all subsequent tests it was reduced by 100%. Neither water immersion nor shampooing the treated dogs affected the efficacy of fluralaner [312]. Crosaz et al. found that oral doses of fluralaner resolved >90% of the cases of FAD in dogs and the FAD clinical scores were reduced by 99.8% at day 168 [313].

A fipronil/permethrin combination provided 95.3% kill against adult fleas 36 h after treatment and 100% after flea challenges up to 8 weeks. The combination prevented flea egg laying from treated pets for at least 57 days [314]. The combination of fipronil/permethrin provided >95% kill of *C. felis* in 2 h for up to 14 days [315]. Studies by Magalhães [316] corroborated earlier findings that the macrocyclic lactone, ivermectin, was not effective in controlling *C. felis* adults [377].

Dogs treated and held with access to the outdoors were treated monthly for 4 months with dinotefuran/pyriproxyfen/permethrin, fipronil/methoprene, and imidacloprid/permethrin. All 3 provided >95% kill for at least 116 days. There was no flea egg production for 120 days with any of the treatments [317].

Imidacloprid/flumethrin collars on dogs provided 31.7 to 64.8% efficacy from day 135 to 232 whereas monthly fipronil/methoprene treatments provided >95% kill. In this study, the dogs were exposed to a water shower every other week to simulate rainfall [318]. In contrast, an imidacloprid/flumethrin collar on dogs provided 94.5–100% kill of fleas for at least 191 days where as a deltamethrin collar was significantly less effective at each test period. There were no differences in efficacy between the imidacloprid/flumethrin collar and fipronil/methoprene or dinotefuran/pypriproxyfen/permethrin spot on applications at days 163–198 [319]. An imidacloprid/flumethrin collar on dogs killed >97% of fleas within 24 h for 105 days and 90–98% kill from day 97 until 217. The dogs were immersed in water or shampooed 1 week prior to testing fleas without any effect on the efficacy. A blanket in contact with the collared dogs provided >99% of flea larvae for 35 weeks [320]. Similarly an imidacloprid/flumethrin collar provided >95% reductions of fleas on cats for 230 days [321]. An imidacloprid/flumethrin collar provided 98–100% kill of fleas for 8 months on cats. Monthly applications of fipronil/methoprene provided 68–99.9% reductions over the same 8 months [322]. An imidacloprid/flumethrin collar on cats provided 99.9% kill of fleas infected with *D. caninum* metacestodes on day 1 [354]. One of the 16 cats acquired 2 scoleces in the treated group, a reduction of 99.7% compared with scoleces acquired in the controls. A similar study conducted with dogs found that the collars provided >99.9% kill of fleas throughout the study [355]. Two of the 8 dogs waring collars were infected with 1 scoleses which was a 96.6% reduction compared with the controls. Imidacloprid/flumethrin collars reduced flea populations on cats and prevented the transmission of *B. henselae* for 8 months [356].

A combination of fipronil/methoprene/cyphenothrin applied to dogs provided >98% kill of fleas when fleas were counted at 24 h for 4 weeks. The strain of *C. felis* (KS1) tested is reported to have reduced susceptibility to fipronil and pyrethroids suggesting that the combination may have some synergistic activity [351]. A combination of fipronil/methoprene/epinomectin/praziquantel applied to cats to control five different strains of *C. felis* provided 94.3% efficacy at 24 h and >95.9% kill when fleas were counted at 24 h 5 weeks later. The treatment reduced the emergence of new fleas for at least 5 weeks [388].

To determine the intrinsic activity of spinetorum and spinosad, adult *C. felis* were exposed in the bottom of vials with short dog hairs treated with serial dilutions of each insecticide. Spinetoram (LC_50_ = 0.724 ng/cm^2^) was about 4–5 times more active than spinosad (LC_50_ = 2.791 ng/cm^2^). A single oral dose of 60 mg/kg or three 20 mg/kg doses of spinetoram to dogs provided >99% kill of fleas for 72 days [389]. A topical application of spinetoram on cats provided >95% kill for at least 37 days [323].

An oral afoxolaner/milbemycin oxime provided dogs killed 100% of the fleas at day 35 and prevented dogs from acquiring *D. caninum* infestations where as 70% of the dogs in the control acquired tapeworms [390].

#### 5.1.3. Effect of Active Ingredients on Feeding

In recent years, more attention has been focused on how fast treatments kill adult fleas. One of the prevailing thoughts has been that the speed of kill of adulticides is important in the prevention of FAD and providing control [175]. Siak and Burrows state, “… rapidly reducing the total flea numbers and decreasing flea feeding time are the keys to controlling the clinical signs of FAD” [216]. However, as Dryden points out that 89% and 92% of the fleas fed on imidacloprid and fipronil treated cats, respectively, even though all the fleas were ultimately killed [391]. Later research has shown that even systemics may have positive effects on relieving symptoms of FAD. If the treatment kills fleas before eggs are laid or if it has ovicidal effects on the eggs, then it will help suppress the flea population within structures [362].

Even after topical applications or oral doses to pets, flea feeding on the host may still occur. Topical applications of imidacloprid and fipronil did not prevent fleas from feeding within the first hour whereas topical applications of dichlorvos/fenitrothion and permethrin decreased feeding by 80% [392]. Significantly more blood was consumed by fleas feeding on imidacloprid and fiprionil treated cats than on cats dosed with nitenpyram or selamectin [288]. McCoy et al. write “These data therefore suggest that systemically acting flea control products may be more effective in interfering with flea biting and feeding than are products whose action is solely topical” [288].

Fipronil applied to dogs provided 74%, 94%, and 100% kill of *C. felis* at 6, 12, and 18 h, respectively [289]. Within 1 h of an oral dose of nitenpyram 38% of fleas were dislodged and 100% were killed within 4 h [301]. Nitenpyram provided >99% kill of fleas within 3 h and 100% kill within 8 h on both dogs and cats. At 8 h on cats, cythioate, imidacloprid and fipronil killed 97%, 83%, and 63% of the fleas, respectively. At 8 h on dogs, selamectin, imidacloprid and fipronil killed 74%, 96% and 47%, respectively [301]. Initially, topical applications of selamectin, imidacloprid, and fipronil/methoprene on cats provided 0%, 87%, and 29% kill at 6 h, respectively. When fleas were counted at 24 h, all three treatments provided >96% kill for at least 21 days [290].

Topical application fipronil/methoprene provided faster kill of fleas compared with metaflumizone for up to 24 h. Metaflumizone provided >95% kill when fleas were counted at 24 h for 14 days and fipronil provided >95% kill for 42 days [324].

Topical applications of dinotefuran and imidacloprid on cats provided 100% and 99% kill at 6 h, respectively. When challenged at day 29, dinotefuran and imidacloprid provided 95% and 57% kill of adult fleas at 6 h, respectively [385].

Topical applications of selamectin provided >98% reductions in adult fleas between 24 to 36 h on dogs and 12 to 24 h on cats [280]. Spot-on applications of selamectin and fipronil/methoprene to cats provided >95% kill of adult fleas and reduction in flea egg production for 38 days with only a few flea eggs hatching [291]. A topical application of fipronil/methoprene on dogs killed 100% of fleas within 12 h for at least 21 days and >99% for up to 28 days [325]. A topical application of pyriprole killed >95% of the fleas within 12 h [384].

The mortality of fleas, counted 6 h after placing them on cat treated with imidacloprid, selamectin, fipronil/methoprene, and metaflumizone was 61%, 47%, 19%, and 8%, respectively [292]. After 28 days, imidacloprid, selamectin, fipronil/methoprene, and metaflumizone provided 28%, 31%, 60%, and 59% kill, respectively, when counted at 2 h.

A topical application of dinotefuran/pyriproxyfen/permethrin on dogs provided >95% kill when counted at 2 h for at least 30 days [317]. Within 5 min 11.2% of the fleas on dogs treated with dinotefuran/pyriproxyfen/permethrin were dislodged compared with 0.2% for animals dosed with spinosad [343]. At 1 h, 54.9% of the fleas were dislodged on dinotefuran/pyriproxyfen/permethrin treated dogs. Real time quantitative PCR showed that there was an 89.3% reduction in feeding within 5 min and this lasted for 30 days.

Two isoxazoline compounds, afoxolaner (monthly doses) and fluralaner (single dose), administered orally provided 100% efficacy for 90 days [363]. Dogs treated with afoxolaner 1 day prior to infestation killed 100% of the fleas within 6 h [361]. Afoxolaner on dogs provided >95% kill within 8 h [393]. When tested for residual activity, afoxolaner provided 97% kill of fleas collected after 6 h from dogs. It decreased to 73.3% at day 28. Fluralaner provided significant reductions of adult fleas within 2 h after dogs were dosed [367].

A soft chewable tablet containing imidacloprid given to puppies and adult dogs provided 96% kill of fleas at 4 h [368].

Sarolaner provided >95% kill of fleas within 8 h of challenging dogs dosed with 2 mg/kg sarolaner for at least 35 days [307]. An oral dose of 50 mg/kg of an isoxazoline benzoxaborle (AN8030) to dogs provided 100% of fleas for 32 days [394]. A combination of selamectin/sarolaner applied to cats provided 72.5% kill within 12 h on day 1 and the kill increased to 93.8% when tested at 28 days. When counted at 24 h, the combination produced >98% kill for at least 28 days [395].

At day 28, spinetoram, fipronil/methoprene, and imidacloprid provided 51.8%, 33.5% and 24.1% kill within 1 h after applying fleas to treated cats [390]. When counted at 12 h, spinetoram, fipronil/methoprene, and imidacloprid provided 88.8%, 71.7% and 80.8% kill, respectively. A combination of fipronil/permethrin provided 69.6% kill of fleas within 0.5 h on dogs treated 8 days earlier [396].

In spite of all the data regarding the speed of kill provided by various products, the following simulated home environment and clinical field studies suggest it is more important to provide residual protection that kills and prevent fleas from feeding and laying eggs. Differences in the speed of kill are of secondary importance.

### 5.2. On-Animal Studies in Simulated Home Environments

Simulated home environments have been used as models to determine the effectiveness of on-animal treatments to control fleas on both companion animals and in the indoor environment. Therapies that effectively control flea populations in these types of studies indicate that additional environmental insecticide applications are not required to provide control of indoor flea populations [293].

Fenthion, lufenuron and a combination of fenthion/lufenuron on cats provided a 91.3,% 72.3%, and 98.6% reductions, respectively, in the number of fleas counted at day 50 [397]. Dogs given lufenuron had the number of fleas ranging from 9–14 fleas/dog at day 28 which declined to 0–0.9 fleas/dog at day 90. On cats, there were 9 fleas/cat at day 28 and there was >95% reduction at day 90 [398]. Nitenpyram, lufenuron, and combination treatments were evaluated by Cadiergues et al. [399] with the addition of lufenuron/nitenpyram resulting in >94% kill of fleas for at least 84 to 112 days.

Imidacloprid and fipronil provided 100% control of adult fleas on cats within 30 days, but lufenuron treatments required some additional dichlorvos/fenitrothion to provide control [244]. Monthly treatments of imidacloprid and lufenuron provided 100% and 66% reductions, respectively, in adult flea numbers compared to the control for 112 days [400]. A topical treatment of fipronil/methoprene provided >99.1% kill of adult fleas for 42 days, but weekly shampooing decreased its efficacy to 34.8% by day 42 [326].

Monthly spot-on applications of selamectin provided >99% reductions of fleas on dogs and cats for the entire 3-month study [293]. Monthly topically treatments of selamectin and fipronil on cats provided 35.3% and 71.2% reductions at day 14, respectively. By day 28, both treatments provided >98% reduction of fleas [294]. A monthly treatment regimen of selamectin provided 99.9% reduction in the number of adult fleas on dogs [282]. A similar regimen of lufenuron provided 45.9–93.5% reductions, but never achieved 95% kill of the adult fleas. The origin of the adult fleas on the lufenuron treated animals was not determined. Over the last 2 months of the study, 20 additional fleas were placed on the dogs or cats monthly. Both treatments continued to prevent larval development even after the last dosage at 120 days.

In a simulated study, a topical application of pyriprole provided 100% reductions of fleas for 60 days [384]. The combination of imidacloprid/pyriproxyfen provided flea free dogs in simulated home studies within 56 days [335]. The combination provided faster and more complete elimination of adult fleas than did the adulticide spinosad.

Dogs were treated monthly with dinotefuran/pyriproxyfen/permethrin, fipronil/methoprene, or imidacloprid/permethrin and held outdoors. All three treatments provided >99% reductions in adult fleas for at least 166 days. No eggs were collected for 120 days during the study [327].

No fleas were collected from cats dosed with spinosad from day 15 to 90. Cats were evaluated for scored for FAD and their scores were reduced by 98% by day 90 [401]. Oral doses of fluralaner provided >99% control in a simulated home environment for 12 weeks [366]. In a simulated home environment, >95% reductions of fleas occurred within 2 weeks and fleas were eliminated after two monthly doses of sarolaner. Greater than 95% kill of fleas occurred within 4–8 h for at least 28 days after animals were dosed [309].

### 5.3. On-Animal Studies in the Field

Two types of field studies are typically conducted. The most common type of study is to enlist pet owners at clinics and provide treatments to their pets. The pets are then regularly evaluated at the clinics. A second type of study involves treating the pets at the clinic, and then monitoring flea populations on the animals and within homes. In a number of these studies, the animals were also evaluated for FAD at the beginning and the end of the study.

A total of 294 dogs and 296 cats were treated with nitenpyram or nitenpyram/lufenuron daily for two weeks. In both groups, nitenpyram given daily for 2 weeks provided >95% kill of adult fleas on dogs and cats [300].

In Italy, 3272 cats and dogs were treated with imidacloprid in clinics. There was a 90–96% reduction in the number of fleas on dogs and a 90–95% reduction on cats over the 4-week study. There was a 20% increase in the number of cats and dogs without FAD [402].

In Georgia, 42 cats from clinics were treated with a topical spot-on fipronil. There was a 94% reduction in flea counts at day 90 and pruritus was reduced or eliminated in 78% of the cats [403]. In a study with 31 dogs with FAD, fipronil spot-on provided a 98% reduction in flea numbers and an 84% reduction in pruritus by day 90. The dermatological scores significantly improved [404].

In clinics across the US, 220 dogs and 189 cats were treated with selamectin. Monthly treatments provided >95% kill of fleas on cats and dogs at day 60 and 90 and there were improved clinical signs of FAD. In comparison, topical applications of fenthion on dogs provided 72–86% reductions of fleas and pyrethrins applied to cats provided 55–83% reductions in fleas over the 90-day study [296]. In clinics across Europe, 191 dogs and 182 cats were treated with selamectin topically and 93 dogs and 86 cats were treated with fenthion. Selamectin reduced fleas on dogs by 92.7–98.4% and 92.7–98.4% on cats over the 3-month study. Fenthion reduced the number of fleas by 80.5–93.8% in dogs and 72.6–87.5% in cats [297].

Spinosad was given orally monthly to 113 cats and selamectin was applied topically each month to 71 cats in 18 clinics in Germany and Italy. Both treatments provided >97% reductions of cat fleas within 14 days and >98% reductions at day 60 with significant improvement in the FAD animals [344]. Selamectin and spinosad were evaluated in a clinical trial of 470 flea infested dogs in the US and Canada. At day 15, spinosad provided 98.6% reduction in flea counts compared with 90.0% for selamectin. At day 90, both treatments provided >98.9% reductions in *C. felis* [345]. Similarly, a clinical trial with selamectin and spinosad resulted in >95% reductions in flea counts at day 90. At day, 85% of the dogs treated with spinosad were flea free compared with 67% of dogs treated with selamectin [295].

In another study, spinosad reduced flea numbers by 99% at day 90 compared with 88% with fipronil/methoprene. At day 90, 94.8% of the dogs given spinosad were flea free compared with 38% in the fipronil/methoprene treatment. At day 90, there was a positive 95% and 49% improvement in FAD scores for spinosad and fipronil/methoprene, respectively [329]. Cats were provide oral spinosad or topically treated with selamectin, vomiting occurring in 14.3% and 2.4% of the cats, respectively. When cats were treated for 3 consecutive months, both treatments provided high levels of control 97–99%. At day 85–95, 93% of cats were flea free when treated with spinosad and 64.7% were flea free when treated with selamectin [346].

Topical applications of pyriprole or fipronil/methoprene applied to each of 6 dogs for 3 months provided 94.6–100% and 81.2–98.8% reductions, respectively, over 90 days [369]. In another study, a total of 233 dogs and 180 cats from 21 clinics in 7 European countries were enrolled. Initially, 41.6% dogs and 47.2% of cats were infested with *C. felis*. Monthly applications of fipronil/methoprene resulted in 91.7% and 89.4% of dogs and cats, respectively, being flea free at day 90 [330]. The efficacy of a spot-on application of imidacloprid/permethrin applied to 62 dogs was evaluated over 11 months with dogs being flea free for 8 months. In late summer, the percent dogs infested climbed to 15.2%, but declined to 0% in October [336]. In a field study 229 dogs were treated with imdiacloprid/permethrin and 134 with fipronil spot on applications, both treatments provided >95% flea control for 14 days and >90% control for 28 days [337].

Dogs diagnosed with FAD were treated with oral doses of fluralaner and evaluated for fleas and FAD over 168 days. The flea counts were reduced to 0 by day 28 and remained there for 168 days. More than 90% of the dogs showed complete resolution of FAD by the end of the study [313]. There was >99.7% reduction in fleas of dogs dosed with a single fluralaner tablet for at least 12 weeks. Three monthly doses of spinosad and an amitraz collar provided >96.5% reduction in flea counts for at least 12 weeks [357].

A trial conducted at 19 clinics throughout the US involved 186 and 94 dogs being treated with oral doses of sarolaner or spinosad monthly for 3 months, respectively [358]. Sarolaner provided >99% kill of fleas and spinosad provided 90 to 98% kill of fleas over the 90 day trial. Both treatments provided substantial improvement of all clinical signs of FAD. Within 2 weeks, 81–88% of the dogs were flea free. In a pharmacokinetic study, a soft chewable tablet containing imidacloprid given daily for 14 days provided 98% kill of adult fleas [368].

In the following clinical field trials, in addition to examining the pets, homes were also monitored for flea infestations. Pets were treated with a topical application of imidacloprid monthly or monthly oral dose of lufenuron and occasional pyrethrin sprays on the pet for 90 days. Both strategies provided >98% reductions of fleas on pets and >99% reductions of fleas trapped indoors at day 90 [359]. In a similar type of study, dogs and cats were topically treated with fipronil or imidacloprid monthly for 90 days [405]. Both treatments provided >95% reductions for the 3 months. Number of fleas in the homes declined by >98% with both treatments. The efficacy of a fipronil/methoprene spot-on application was evaluated in homes in Tampa, FL. Of the 2241 fleas collected in flea light traps, 771 (34.4%) had fed, but only 9 were considered fully engorged. At day 30 there was a 92.5% reduction in the number of fleas trapped. At day 60, there was an 87.5% reduction in the on-animal counts. The continued presence of a few fleas on the pets was attributed to other untreated visitor pets or feral animals in the environment [331].

Topical applications of dinotefuran/pyriproxyfen, dinotefuran/pyriproxyfen/permethrin, or fipronil/methoprene on naturally infested pets in homes provided about 88% reductions in fleas at day 7. All treatments provided >95% reduction in the flea burdens on pets at day 30 and 60. By week 6 the flea trap counts had decreased by 97%. In this natural home environment, it was noted that a few fleas were still encountered even in the most successful treatments [332].

A monthly application of indoxacarb to dogs provided >95% reductions for at least 60 days. Topical applications of fipronil/methoprene provide between 49 and 85% kill of fleas over the study [333]. Indoxacarb reduced the number of fleas trapped by 72–98% and fipronil/methoprene reduced the numbers by 60–85%. Dryden et al. suggest, “The reduced efficacy of the fipronil formulation could be the result of resistance, innately tolerant flea strains or potentially other factors as yet unknown” [333].

Monthly applications of a spot-on fipronil/permethrin combinations provided >96% mortality of *C. felis* and *canis* for 84 days [80].

A chewable formulation of afoxolaner given to dogs provided >99% reduction in the number of fleas on dogs within 7 days. A second dose at 1 month completely eliminated the fleas on the dogs. There was a significant reduction in the number of fed fleas captured with light traps by day 14. Of the fleas captured only 4.9% had fed on a host [362].

### 5.4. Alternative Treatments

Pet collars have been widely marketed for years as an alternative to sprays, dips and shampoos. Witchey-Lakshmanan [406] provides a review of the technology behind insecticide impregnated collars and discussion of their advantages such as their ease of use and potential for long-term control. Despite their availability for decades little data has been published on the efficacy collars until recently. Methoprene impregnated collars reportedly provided 98% reductions in adult fleas on pets over a 6 month study [407]. Collars with 1% methoprene provided >94% inhibition of egg hatch for at least 184 days on dogs and collars with 2% methoprene provided 94–100% inhibition of egg hatch for 365 days on cats [408]. After 14 days, collars containing either deltamethrin or diazinon provided >95% reductions of fleas for at least 91 days. After 91 days, the performance of the diazinon collar decreased dramatically [409]. The combination of imidacloprid/flumethrin incorporated into a collar provided for repellency of ticks and the kill of adult *C. felis* for up to 34 weeks. On 271 dogs, imidacloprid/flumethrin collars provided 96.7% control over 8 months whereas a dimpylat (diazinon) collar provided 79% and 57.9% control in cats and dogs, respectively [410]. The active ingredients dispersed to bedding materials provided >90% control of immature stages of *C. felis*. A collar containing imidacloprid/flumethrin provided 100% control of *C. felis* for 8 months on dogs under field conditions [411].

Initially 55 and 60 dogs in an outdoor/indoor kennel were fitted with an imidacloprid/flumethrin and deltamethrin collars, respectively. At the beginning of the study, 23.6% of the dogs fitted with imidacloprid/flumethrin collars were initially infested with fleas and none were infested with fleas at 4 and 7 months. On the first day, 10.0% of the dogs fitted with the deltamethrin collars were initially infested and at the end of 4 months 33% were infested. There was a 83% reduction in *Lesihmania infantum* infections with dogs with imidacloprid/flumethrin collars and a 61.8% reduction with deltamethrin collars. The imidacloprid/flumethrin collar provided 98.3% overall control of fleas over 8 months on 232 cats tested [412].

CatanDog’s^®^ tags (a non-chemical and non-toxic tag) failed to control fleas on pets and had no effect on egg production or egg viability [413]. Essential oils such as limonene have been used in the past to control adult cat fleas. Essential oils and extracts from the Brazilin peppertree, *Schinus molle*, were toxic to adults, but not to the eggs. Non-polar fractions including compounds myrtenal, terpineol, spathulenol, cubenol and lupenone may have potential as adulticides [414]. An oral dose of powdered aloe juice administered to dogs had no effect on adult cat fleas [415].

Nisbet [137] provides a review of the development of flea vaccines. The lack of a natural immunity to flea infestations by companion animals has been an obstacle to developing a vaccine. The focus has turned to the discovery of molecular targets of expressed sequence tags. Vaccines may be useful in limiting the accumulation of large number of fleas in the environment [210].

### 5.5. Hosts Other than Cats and Dogs

Occasionally *C. felis* is a pest on animals other than cats and dogs presenting an unusual control problem. Populations of *C. felis* were established on ferrets and topical applications of imidacloprid at 10 mg/kg body rate provided >95% reduction at 8 h and 100% reductions at 24 h. However, the residual activity provided <95% kill between weeks 1–4 [416]. A dose between 20–50 mg/kg of imidacloprid topically applied to ferrets provided >95% kill for 23 days [417].

A variety of zoo animals including white wolves, beech-marteens, raccoons, and coatis were infested with fleas and treated with lufenuron. The infestations and dermatitis were resolved with 3 to 6 weeks [418].

A combination of imidacloprid/permethrin provided 100% kill of *C. felis* on naturally infested rabbits [419]. Dairy calves have been reported as sources for *C. felis* infestations in Brazil [420,421]. A severe infestation of *C. felis* on dairy calves in Brazil was treated with a 1.0% fipronil pour-on [421] and another with imidacloprid [420].

## 6. Environmental Control

Since the development of the on-animal therapies, less attention has been given to environmental treatments. There has been an increased awareness of feral animals serving as a reservoir for *C. felis* populations and the possible need to treat outdoors. Alternative strategies to controlling fleas include biological control, sanitation, and environmental modifications [406,422]. Some treatment strategies have been reviewed [10,174]. However, very few pet owners (<5%) attempt to control fleas in the environment [142].

The treatment of the indoor environment has dramatically declined with the increased use of on-animal therapies. Carpets provide a substrate for *C. felis* to pupate with nylon and wool loop carpets providing greater protection from pesticide sprays than do nylon Saxony and nylon contract carpets [261]. At the tuft base of carpet pupae are protected and this remains a weak link in household flea control. All stages of cat fleas including the adults and pupae are killed by vacuuming [423]. Vacuuming removed 40–80% of eggs in the carpet, but only 5% of the larvae [122]. Length of carpet fibers closely associated with the effectiveness of removing immature stages. Overall vacuuming had a limited effect in this study. Clearly, operational factors such as the carpet and vacuum cleaner make a difference in the level of control.

A volatile silicone based material, 0.4% dimeticone spray, immobilized larval and adult fleas and inhibited the emergence of adult fleas [424]. When applied to carpet, it provided comparable activity to permethrin and pyriproxyfen for 3 weeks.

Carpets can be contaminated with ectoparasiticides by direct contact with treated pets or by the accumulation of debris from treated pets. Cats treated topically with imidacloprid were confined to pieces of carpet for 1 or 6 h. The exposed carpets were tested at day 1–2 and the adult cat flea emergence was reduced by 81–82%. The imidacloprid’s residual activity declined by day 29 providing only 33% kill of larvae [425]. Similarly, blankets from cages with cats treated with imidacloprid prevented eggs and larvae from developing into adults [263]. Blankets aged for 18 weeks reduced adult emergence by 94.7–97.6%. Washing and low temperature tumble drying destroyed the insecticidal activity of imidacloprid on the blankets.

Surfaces have also been treated with IGRs and pyrethroids to control immature stages. The LC_50_’s for the IGRs methoprene and pyriproxyfen applied to top soil against larvae were 0.643 and 0.028 ppm, respectively [426]. Pyriproxyfen applied to wood surfaces at 16–32 mg (AI)/m^2^ completely inhibited larvae from developing into adults. A combination spray of cyfluthrin/pyriproxyfen applied indoors inhibited 99.9% of the immature stages of *C. felis* from developing for 18 weeks [427].

## 7. Toxicology of Ectoparasiticides

There are a number of reviews of the toxicology of insecticides used to control fleas on small animals [220,428,429,430,431]. The pharmacology and therapeutic use for flea control of the insect nicotinic acetylcholine receptor agonists, imidacloprid, nitenpyram, dinotefuran, and spinosad, were reviewed [428,431].

A review of the use and misuse of pyrethroids against cat fleas indicate a high incidence of feline toxicosis after off label use of topical formulations [429]. Anadón states, “ In most cases, misuse by the animal’s owners or accidental ingestion (by mouth or grooming) of commercial preparations such as collars, powders or sprays containing such compounds for use in flea control have been the cause of poisoning by increasing toxicity” [429]. Combinations of pyrethrins and pyrethroids with synergists such as piperonyl butoxide, organophosphates or carbamates may increase their toxicity. Of the 750 cases over 2 years of permethrin spot-on intoxication reported in Australia all but one were the result of using a product labelled for dogs only [432]. In Australia, 41 of 42 cases of permethrin toxicity in cats was the result of a permethrin spot on product manufactured for dogs being misused. Survey data suggests that most cases were the result of over-the-counter products [433]. In Canada from 2007 to 2009 there were 708 companion animal incident reports involving flea and tick control products of which 14 posed life-threatening symptoms. About 5% (236) responded and half of them felt the most important factor contributing to problems in cats was the misuse of dog products on them. Another suspected factor was the accidental transfer of products between animals [434]. The US EPA reported that the misuse of dog products on cats was an important problem and recommended some changes in regulating spot-on products, reporting data on pet incidents, and labeling on packages [435]. Education of the pet-owner is important to prevent accidental exposures of cats to pyrethroids [436].

From 1989–1997 there were 16 cases of pesticide-related illness due to occupational use of flea control products including flea dips and shampoos [437]. There is a concern that individuals repeatedly handling treated pets have greater risk of exposure. Several studies to determine the amount insecticide that is bioavailable from the pelage of treated animals have been conducted. Cotton gloves are used to sample the pelage and the amount of pesticide that might penetrate the skin is not known. Transference of fipronil to cotton gloves from dogs treated with Frontline was greatest at day 1 and week 1 and then gradually declined [438]. The highest exposure of selamectin was within first 24 h [439] and repeated exposure could pose potential health threats to veterinarians, vet technicians dog/trainers/handlers and pet owners. Ten repetitive petting simulations with cotton gloves of dogs treated with indoxacarb resulted in maximum 2% transfer. The initial 2% transfer on the day of treatment declined to 0.08% at day 30 [440].

Hair clippings of brushing of dogs treated with fipronil spot-on had <10% of the fipronil applied to the animal [441]. Low levels were found on human gloves after petting the animals. Urine biomonitoring revealed that human exposure to fipronil is low. Residues of tetrachlorvinphos (TCVP) on gloves used to rub dogs with collars containing TCVP were 16,600 μg/glove and 1.8 μg/g of tee shirts worn by children contacting the dogs [442]. The lack of cholinesterase inhibition in dogs and the low acute toxicity of TCVP suggest that it is rapidly detoxified and excreted. Using gloves to contact treated pets, the highest risk of transference of imidacloprid was at 12–24 h after application [443]. The imidacloprid residue persisted up to 4 weeks, but no definitive conclusions were reached concerning its potential health impact.

The US EPA reported concerns for potential human exposure to pet collars containing TCVP and is working with manufacturers to address these risks [444]. Little if any additional exposure to chlorpyrifos residues was found in dogs wearing chlorpyrifos pet collars [445]. TCVP and chlorpyrifos collars were tested for transferable residues. There was no definitive statement regarding the risk assessment [446]. The concomitant use of the pet collar containing imidacloprid/flumethrin and spot-on applications of imidacloprid/moxidectin in dogs and emodespside/praziquamtel did not affect the collars or reveal any significant dermal findings or systemic safety findings [447].

Dogs dipped in chlorpyrifos preparations had the maximum inhibition of butyrlcholinesterase at day 7. The greatest risk of human exposure was shortly after dipping with the largest decrease of transferable residues occurring within the first 7 days [448]. Dogs dipped in phosmet resulted in no serum cholinesterase inhibition suggesting that there is either very low dermal absorption or there was rapid detoxification. There was no correlation between hair length of the dog and the amount of transferable residues. The transferable residues dropped 62% after the first day [449]. Insecticidal shampoos, dips and collars containing chlorpyrifos may expose humans to low levels of insecticide [450].

Relatively few papers concerning the effects of insecticides on companion animals exist in the literature. There was no increased risk of transitional cell carcinoma in Scottish terriers after topical applications of fipronil or imidacloprid [451]. Nitenpyram caused a 3–6 fold increase in flea-related itching in cats and dogs, but this was attributed to affected fleas and not the product [452]. Shampoos containing *d*-limonene on cats can cause acute necrotizing dermatitis and septicemia [453]. Outdoor applications of diazinon granules to lawns exposed dogs to greater levels of diazinon than to the occupants and dogs served as a good vehicle to uptake, transfer, and translocate pesticide residues indoors [454].The relationship between contacting treated pets by humans and transfer of insecticide residues was unclear.

The US EPA has recommended that pet owners use caution when using spot-on pesticide applications to pets [455].

## 8. Treatment Failures and Insecticide Resistance

Since 1997, there have been a number of reviews regarding insecticide resistance in *C. felis* [3,213,456,457,458]. To date, there has been limited evidence of insecticide resistance developing to any of the numerous on-animal or oral therapies. In one case, cats were treated with fipronil spot-on and challenged with a susceptible strain of cat flea and a field-collected isolate that had an LD_50_ Resistance Ratio of 26 by topical application. A 24-h exposure to fipronil treated cats provided >96% kill of susceptible fleas and only 32.6% kill of the fipronil resistant fleas when the treatments had aged 28 days [459]. A second possible example was reported from a field-collected strain of *C. felis* that had low sensitivity to imidacloprid in contact exposure tests on filter paper compared with more susceptible isolates of *C. felis* from three other dogs [460]. To date these are the only reported instances of insecticide resistance to the modern on-animal and oral treatments. The general consensus is that reports of reduced performance are largely attributed to operational aspects and treatment deficiencies [213,460,461,462].

A number of operational factors may be responsible for the apparent decline in control of fleas. Feral animals such as opossums, raccoons or feral cats can serve as source of fleas outdoors. Halos et al. cited external flea sources, both from the environment and feral hosts, as one of the reasons for persistent flea problems and the lack of control [461]. In some situations humans can transport fleas indoors to pets [462]. Failure to adequately treat and follow label directions can contribute to continuing problems. Lastly, the failure to treat all the pets within the household is an issue [213].

To determine the esterase activity, including glutathione-S-transferase and cytochrome P-450 monooxygenases (responsible for resistance to organophosphate insecticides), a rapid assay was developed [463]. However, with the phase out of carbamates and organophosphates in the late 1990s in the US, this rapid assay has not been conducted on many field-collected isolates.

A laboratory strain of adult cat fleas was topically treated with 13 different insecticides and toxicities ranged from nitenpyram (0.68 ng/flea) to carbaryl (>10,000 ng/flea) [200]. There was a considerable range of response to four pyrethroid insecticides in 12 field-collected isolates of *C. felis* [464]. When compared with earlier data from Moyses [200], deltamethrin and permethrin showed a level of resistance in all of the field isolates. The LD_50_s of the pyrethroids, bioallethrin, deltamethrin, esbiothrin, and permethrin to a laboratory strain of *C. felis* were 121, 0.38, 161, and 23 mg/m^2^ on filter paper [197]. Both L10154F and T929V mutations are common in both lab and field populations [465]. Mutations (*kdr* and *skdr*) conferring target-site resistance to pyrethroids segregated in opposition to one another, precluding the possibility of genotypes homozygous for both mutations. The mutation A302S conferring resistance to cyclodienes and varying levels of cross-resistance to fipronil was detected in 8 of 9 strains analyzed [466]. Resistance ratios of field-collected strains from Australia and 8 different states in the US to pyrethroids ranged from 1 to 4.9 [464]. These strains were heterozygous for the *kdr* and *skdr* mutations. Many of the strains had been reared in the laboratory for years and not exposed to insecticides. A PCR-based diagnostic assay showed that the A302S mutation existed at high frequencies in fleas collected from clinics in the UK and US. This is especially significant considering the recent increase in combination products being promoted for on-animal treatments containing pyrethroids. The effect or impact of utilizing pyrethroids in these combinations against resistant field strains remains unknown. It certainly warrants additional research attention.

The *rdl* mutation (conferring target-site resistance to cyclodiene insecticide) from cat fleas was sequenced and two PCR based diagnostic tests reported [467]. Topical applications of fipronil to pets provided >95% kill at day 29–30 with 5 of 6 cat flea strains that were homozygous for the *rdl* mutation (Ala^285^ to Ser substation). The data clearly suggests that any recent decline in activity of fipronil was not due to the *rdl* mutation [468].

The Flea Susceptibility Monitoring (FSM) program sponsored by Bayer Animal Health has monitored the insecticidal activity of imidacloprid with field-collected isolates of *C. felis* over the past 17 years [469,470,471,472,473]. *C. felis* eggs were collected by cooperators in the US, UK, France, Germany and Australia and shipped to several laboratories where larval bioassays were conducted [203]. Shipping eggs minimized mortality of fleas in transit and allowed them to be tested upon arrival. A diagnostic dose of 3 ppm imidacloprid in larval rearing media was established to expedite screening [204]. Adult fleas were tested with topical applications of imidacloprid and fipronil and compared with larval bioassays. Both bioassays provided similar data, verifying the larval bioassay [205]. In recent years, the lack of egg hatch in some of the field-collected isolates suggested the possibility that treatments with IGRs on the cats or dogs may have prevented eggs from hatching and the initial larval bioassay procedure was slightly modified. When debris collected with the flea eggs in the clinics was bioassayed with laboratory *C. felis* eggs, about 67% of those samples tested may have had some IGR or other toxic contamination at some point [471]. Over 17 years, more than 1500 isolates have been tested and none of them have shown any decreased susceptibility to imidacloprid. If resistance should appear, Bass et al. write “The identification of cat flea nAChR subunits that have a high affinity for imidacloprid presents candidate genes in which to look for resistance-associated mutations if target-site resistance to imidacloprid arises in domestic pet flea populations” [474].

## 9. Natural and Biological Control

In spite of the increased interest in so-called green pest management, there have been very few advances in controlling fleas with biological agents, natural products or mechanical means. The entomopathogenic fungi *Metarhizium anisopliae* successfully inhibited flea eggs from hatching and *Beauveria bassiana* was successful in killing adult fleas [475]. While pathogenic these fungi have been repeatedly shown to be toxic to fleas, they never been developed in to successful control strategies.

Grooming in cats is an effective means of reducing adult cat flea numbers. In 2–3 weeks, about 41% of the adult fleas were removed by grooming compared with a slight increase in flea numbers on cats with an Elizabethan-collar [476]. Flea infested cats groomed at twice the rate as the control cats. If the flea life cycle can be broken utilizing IGRs or other adulticides, then grooming will certainly help resolve the problem.

Sticky traps with intermittent light caught more fleas than did traps with continuous light [477]. A green filter increased trap catch. Thermal cues apparently had no effect. This has been shown in certain studies to be an effective means of monitoring indoor populations [400,401,402,403,404,405]. However, without additional treatments this alone will not resolve the flea infestation.

## 10. Integrated Pest Management (IPM)

Integrated strategies involve both mechanical and chemical control and are recommended in extreme and desperate situations [210]. On-animal and oral therapies have been shown to be very effective in interrupting the flea life cycle indoors.

A model has been designed to evaluate integrated control measures against cat fleas. It accounts for the biological and chronological characteristics of fleas. The model confirms the resistance to treatment of the cocoon stage and the need to use persistent applications to the pets [478]. Otranto et al. propose that more IPM approach to flea control including the development of vaccines, models of population dynamics, and trapping [146].

The treatment of feral animals and outdoor populations of *C. felis* remains problematic. Existing pyrethroid sprays provide marginal control because of widespread resistance. Other active ingredients such as fipronil, metaflumizone, and selamectin are not registered for premise control. This has presented a challenge for pest management professionals when encountering outdoor infestations associated with feral animals.

Cat fleas can also be a problem in residences and buildings in which there are no companion animals. Extensive vacuuming and trapping may help remove adult fleas, and the use of space sprays is extremely limited.

## 11. Conclusions

Since the last reviews of the biology and control of cat fleas, there have been numerous advancements in our understanding of the biology of the cat flea. In most climates, the prevalence of cat fleas is seasonal, but adult cat fleas can be found on hosts year-around. Adult female fleas are prolific breeders and as soon as the environmental conditions favor larval development, populations can explode. Thus, preventative treatments should be highly recommended in most regions.

Numerous on-animal and oral therapies are registered for cat flea control and many of them will provide >95% reductions of cat fleas on pets for at least 30 days. Many of them have also been shown to reduce the impact of FAD in sensitive animals when products are applied according to the label directions. Others have been shown to help protect companion animals from tapeworm and other diseases. In general, the speed of kill of adult fleas is not the major issue with most of the current therapies. The most important outcomes after treatment are the residual activity of the treatment and its ability to break the flea life cycle. The simulated field studies and clinical studies demonstrate that even the slower acting ectoparasiticides are effective. Thus, even oral therapies that require fleas to feed on the treated animals are effective in reducing flea populations, FAD and preventing disease. Field studies have shown that on-animal treatments dramatically impact the populations of fleas and can provide significant reductions in the number of adult fleas in the indoor environment.

In recent years, there has continued to be a total reliance on old therapies and the newly registered insecticides to control fleas. Insecticide resistance to pyrethroids is increasing and widespread. Insecticide resistance to the IGRs or new chemistries has not occurred, but continuous monitoring is adviseable. In the event of resistance, the current arsenal of products to control fleas represents a number of different chemical groups and modes of action that should effectively counter its development. There has been little research conducted on the control of outdoor flea populations, especially on feral animals. This remains an important void in our efforts to control the cat flea and develop a comprehensive IPM program.

## Figures and Tables

**Table 1:** Summary of *C. felis* hosts other than cats and dogs ^a^.

Species (Colloquial Name, *Scientific name*)	Region(s)/Countries	Comments	Key References
African pygmy hedgehog *Atelerix albiventris*	Tanzania	2nd most prevalent ectoparasite	[28]
Common opossum *Didelphis masupialis*	French Guiana		[29]
Domesticated ass *Equus asinus*	Israel	severe anaemia	[30]
Domesticated sheep *Ovis aries*	Israel, Iran, Ethiopia	Seasonal allergic dermatitis	[31,32,33]
Eastern cottontail rabbit *Sylvilagus floridianus*	United States	in zoo setting	[34]
European hedgehog *Erinaceus europaeus*	Germany	7.9% hedgehogs infested	[35]
Gazellas *Gazelle gazelle*	Israel	in zoo	[36]
Goat *Capra aegagrus hircus*	Eygpt, Iran, Ethiopia		[32,33,37]
Golden cat *Catopuma temminckii*	Thailand		[38]
Gray fox *Urocyon cinereoargenteus*	Mexico		[39]
Grizzly bear *Ursus arctos horribilis*	United States	in zoo	[34]
Least weasel *Mustela nivalis*	Eygpt	serological study	[37]
Maned wolves *Chrysocyon brachyurus*	Brazil	in zoo	[40]
Margay *Leopardus wiedii*	Peru	in zoo	[41]
Marsh deer *Blastocercecus dichotomus*	Brazil	in zoo	[42]
Norway rat *Rattus norvegicus*	China, Eygpt		[37,43]
Raccoon *Procyon lotor*	West Virginia, Virginia, United States		[44,45]
Red fox *Vulpes vulpes*	Virginia, South Carolina, United States		[45,46]
Roof rat *Rattus rattus*	Eygpt		[37]
Rüppel’s fox *Vulpes rueppelli*	Eygpt	serological study	[37]
South American coati *Nasua nasua*	Brazil	urban forests	[47]
Striped skunk *Mephitis mephitis*	Connecticut, United States		[48]
Viriginia opossum *Didelphis virginiana*	United States		[34,45,46,49]
Water buffalo *Bubalus bulalis*	India		[50]

^a^ Linardi and Santos [14] reported 41 species of mammals and 1 bird species in Brazil.

**Table 2:** New active ingredients and combinations tested and registered against *C. felis* in in vitro and in vivo tests.

Active Ingredients	Form. ^a^	Registration ^b^	Status	Test Site ^c^	References
Selamectin	Exp.	1999	Active	LIN	[274,275,276]
				LOA	[277]
	RP			LOA	[278,279,280,281,282,283,284,285,286,287,288,289,290,291,292]
				LOA-SE	[293,294]
				CFT	[295,296,297]
Nitenpyram	RP	2000	Active	LOA	[288,298]
				CFT	[299,300,301]
Fipronil/methoprene	RP	2000	Active	LOA	[290,291,292,302,303,304,305,306,307,308,309,310,311,312,313,314,315,316,317,318,319,320,321,322,323,324,325]
				LOA-SE	[326,327]
				CFT	[328,329,330,331,332,333]
Imidacloprid/	RP	2007	Active	LOA	[334]
Pyriproxyfen				LOA-SE	[335]
Imidacloprid/permethrin/	RP	2007	Active	LOA-SE	[327]
pyriproxyfen				CFT	[336,337]
Metaflumizone	RP	2007	Inactive	LOA	[292,324,338,339,340]
Spinosad	Exp.	2007	Active	LOA	[341]
	RP			LOA	[295,302,328,342,343]
				LOA-SE	[326]
				CFT	[295,329,344,345,346]
Dinotefuron/pyriproxyfen/	RP	2007	Active	LOA	[315,316,318,328,343,347]
permethrin				LOA-SE	[327]
				CFT	[332]
Dinotefuran/pyriproxyfen	RP	2008	Active	LOA	[332]
Indoxacarb	RP	2010	Active	LOA	[348,349]
				CFT	[333]
Spinetoram	Exp.	2010	Active	LOA	[308]
	RP			LOA	[309]
Fipronil/amitraz/methoprene	RP	2011	Active	LOA	[319,348]
Spinosad/milbenycino xime	RP	2011	Active	LOA	[350]
Fipronil/methoprene/cyphenothrin	RP	2011	Active	LOA	[351]
Fipronil/permethrin	RP	2012	Active	LOA	[314,315,352]
Imidacloprid/flumethrin	Exp.	2012	Active	LIN	[353]
	RP			LOA	[318,319,320,321,322,354,355,356]
				CFT	[357,358,359]
Afoxolaner	Exp.	2013	Active	LIN	[306,360]
	RP			LOA	[361]
				CFT	[362,363]
Fluralaner	Exp.	2014	Active	LIN	[360,364,365,366]
	RP			LOA	[308,311,312,367]
				CFT	[313,363]
Imidacloprid/oral	RP	2015	Active	LOA	[368]
				CFT	[368]
Sarolaner	Exp.	2016	Active	LOA	[307]
	RP			LOA	[308,309]
				LOA-SE	[309]
				CFT	[369]

^a^ Form. Formulated. RP = registered product, Exp = Experimental formulation; ^b^ data obtained at https://animaldrugsatfda.fda.gov/adafda/views/#/search; ^c^ LIN = laboratory study in vitro; LOA = lab study on-animal; LOA-SE = laboratory on animal in simulated indoor environment; CFT = clinical field trial.
